# Photon bound state dynamics from a single artificial atom

**DOI:** 10.1038/s41567-023-01997-6

**Published:** 2023-03-20

**Authors:** Natasha Tomm, Sahand Mahmoodian, Nadia O. Antoniadis, Rüdiger Schott, Sascha R. Valentin, Andreas D. Wieck, Arne Ludwig, Alisa Javadi, Richard J. Warburton

**Affiliations:** 1 Department of Physics, University of Basel, Basel, Switzerland; 2 Centre for Engineered Quantum Systems, School of Physics, The University of Sydney, Sydney, New South Wales Australia; 3 Institute for Theoretical Physics, Institute for Gravitational Physics (Albert Einstein Institute), Leibniz University Hannover, Hannover, Germany; 4 Lehrstuhl für Angewandte Festkörperphysik, Ruhr-Universität Bochum, Bochum, Germany

**Keywords:** Single photons and quantum effects, Nonlinear optics, Quantum optics, Solitons

## Abstract

The interaction between photons and a single two-level atom constitutes a fundamental paradigm in quantum physics. The nonlinearity provided by the atom leads to a strong dependence of the light–matter interface on the number of photons interacting with the two-level system within its emission lifetime. This nonlinearity unveils strongly correlated quasiparticles known as photon bound states, giving rise to key physical processes such as stimulated emission and soliton propagation. Although signatures consistent with the existence of photon bound states have been measured in strongly interacting Rydberg gases, their hallmark excitation-number-dependent dispersion and propagation velocity have not yet been observed. Here we report the direct observation of a photon-number-dependent time delay in the scattering off a single artificial atom—a semiconductor quantum dot coupled to an optical cavity. By scattering a weak coherent pulse off the cavity–quantum electrodynamics system and measuring the time-dependent output power and correlation functions, we show that single photons and two- and three-photon bound states incur different time delays, becoming shorter for higher photon numbers. This reduced time delay is a fingerprint of stimulated emission, where the arrival of two photons within the lifetime of an emitter causes one photon to stimulate the emission of another.

## Abstract (web summary)

Measurements on a single artificial atom—a quantum dot—coupled to an optical cavity show scattering dynamics that depend on the number of photons involved in the light–matter interaction, which is a signature of stimulated emission.

## Main

Photons do not easily interact with one another. This property is commonly exploited to communicate over long distances using optical fibres. Interaction between photons is desired, however, for classical and quantum information processing, but requires a highly nonlinear medium. Optical nonlinear processes are employed in a range of photonic applications such as frequency conversion, optical modulation, light amplification and sensing^1–3^. In the limit where the optical nonlinearity is expressive on the scale of a few photons, one can observe quantum nonlinear phenomena, for instance via the optical correlation functions^3–5^. One manifestation of the nonlinearity is the presence of two- and higher-order photon bound states. Photons in these bound states are strongly correlated, such that the likelihood of observing a photon at any one time is fixed, but once one photon is detected, the arrival of another is much more likely than at a random time. We emphasise that photon bound states are distinct from bunched photon states, as photon bound states are quasiparticles that have their own dispersion relation and are eigenstates of the underlying Hamiltonian that describes the nonlinear medium. It has recently been predicted theoretically that the photon-number-dependent propagation velocity of photon bound states can lead to the formation of highly entangled, ordered states of light^6^. Photon bound states have been predicted to exist in a number of systems, such as unidirectional waveguide quantum electrodynamics (QED)^7–9^ and strongly correlated Rydberg gases^10^. In the latter case, experimental observations consistent with their presence have been reported^11–13^. A direct observation of their dynamics is, however, lacking. To observe directly the dynamics of photon bound states, we examine the unidirectional propagation of few-photon wavepackets strongly interacting with a single atom, in practice a semiconductor quantum dot (QD) coupled to a one-sided cavity.

**Fig. 1:**
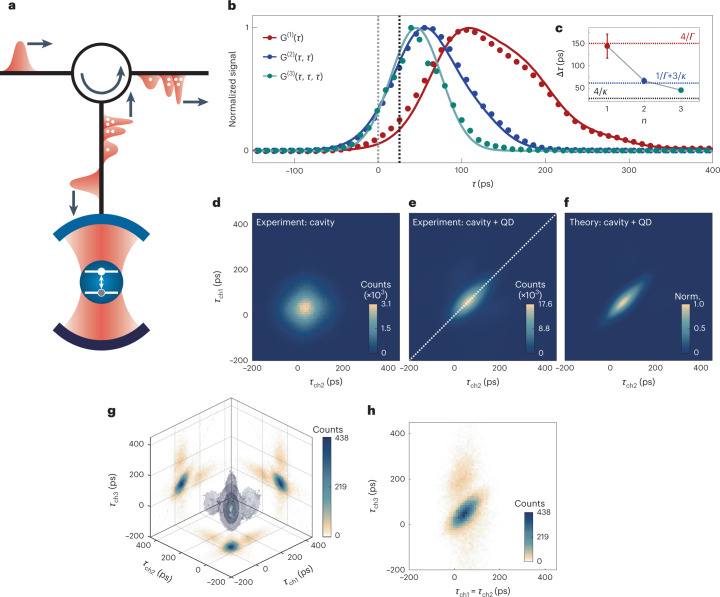
Photon-number-dependent pulse scattering. **a**, A Gaussian pulse of light is launched into a circulator, which guides the pulse towards a QD, coupled to a one-sided microcavity. Upon interaction with the QD–cavity system, states of light with different photon number are transported through the system with different time delays. **b**, Normalised photon counts versus delay. For a Gaussian pulse (*σΓ* = 2.2) launched at time *τ* = 0 (dotted grey line), the propagated pulse undergoes a Wigner delay in the presence of the optical cavity alone (dotted black line). Scattering off the QD–cavity system, the single-photon components *G*^(1)^(*τ*) (red points, experiment; red solid line, theoretical model) undergo pulse reshaping and arrive with a larger delay than the two-photon bound states *G*^(2)^(*τ*, *τ*) (blue points, experiment; blue solid line, theoretical model), which in turn undergo a larger delay than three-photon states *G*^(3)^(*τ*, *τ*, *τ*) (green points, experiment; green solid line, Gaussian fit). **c**, Average pulse peak delay Δ*τ* experienced by *n* photons. Mean and 1*σ* s.d. extracted from 12 (for *n* = 1, 2) and 1 (for *n* = 3) low-power resonant measurements for different input pulse widths. **d**,**e**, Autocorrelation map *G*^(2)^(*τ*_ch1_, *τ*_ch2_) of the pulse following propagation through the entire system in resonance with the optical cavity but in the absence of the QD (**d**) and in the presence of the QD (**e**). The white dotted line represents the equal-time correlation. **f**, Simulation of normalised |*ψ*_2_(*τ*_ch1_, *τ*_ch2_)|^2^. **g**, Autocorrelation map *G*^(3)^(*τ*_ch1_, *τ*_ch2_, *τ*_ch3_). The volumes in the three-dimensional space depict the isosurfaces at 0.05, 0.20, 0.50, 0.75 and 0.90 of the normalised counts, and the projections on each axis are plotted on setting one of the detection times to zero. **h**, Cut-through of *G*^(3)^ at times *τ*_ch3_ and *τ*_ch1_ = *τ*_ch2_. Source data

The experimental set-up is schematically depicted in Fig. 1a. Gaussian pulses of light are guided via a circulator to the one-sided QD–cavity system. The light is back-scattered and redirected by the circulator towards a Hanbury Brown–Twiss (HBT) set-up equipped with single-photon detectors that record the time of arrival *τ* of individual photons. By launching a weak coherent pulse with average photon number ${\bar{n}\ll 1}$, one can probe directly the scattering dynamics of single-photon pulses via power measurements *P*(*τ*) = *G*^(1)^(*τ*), which is proportional to the single-photon wavefunction |*ψ*_1_(*τ*)|^2^. Conversely, the second-order correlation function *G*^(2)^(*τ*_ch1_, *τ*_ch2_) is insensitive to the single-photon Fock component and is used to study two-photon scattering dynamics^14^. *G*^(2)^(*τ*_ch1_, *τ*_ch2_) is proportional to the squared amplitude of the two-photon wavefunction, |*ψ*_2_(*τ*_ch1_, *τ*_ch2_)|^2^. We also measure the third-order correlation function *G*^(3)^(*τ*_ch1_, *τ*_ch2_, *τ*_ch3_) to probe the dynamics of the three-photon component. For higher-order correlation functions, we can determine the equal-time correlators, that is, when *τ*_ch1_ = *τ*_ch2_ = ... = *τ*_ch*n*_. For the coherent state the equal-time correlator is *G*^(*n*)^(*τ*, *τ*, …, *τ*) = *P*(*τ*)^*n*^. Deviations from this indicate that the photons undergo a nonlinear scattering process.

Single-photon states and two-photon states undergo distinct dynamics when scattering off the cavity–QED system. We can understand these dynamics by examining the one- and two-photon scattering eigenstates. In our cavity–QED set-up the QD couples almost perfectly to the cavity, which in turn has small undesired losses (≤5%), and we thus model our system as being lossless. The single-photon scattering eigenstates are plane waves that are transmitted through the system with a transmission coefficient^15–17^

1 $${t}_{1}({{{\varDelta }}}_{{{{\rm{L}}}}},\,{{{\varDelta }}}_{{{{\rm{C}}}}})={\frac{{{{\varDelta }}}_{{{{\rm{L}}}}}-{{{\varDelta }}}_{{{{\rm{C}}}}}-{g}^{2}/{{{\varDelta }}}_{{{{\rm{L}}}}}-i\kappa /2}{{{{\varDelta }}}_{{{{\rm{L}}}}}-{{{\varDelta }}}_{C}-{g}^{2}/{{{\varDelta }}}_{{{{\rm{L}}}}}+i\kappa /2}},$$

where *Δ*_L_ = *ω*_L_ − *ω*_QD_ is the angular frequency detuning of the photon and the QD, *Δ*_C_ = *ω*_C_ − *ω*_QD_ is the detuning between the cavity resonance and the QD, *g* is the atom–cavity coupling, and *κ* is the cavity loss rate. Under the lossless assumption, the scattering amplitude is unitary, |*t*_1_| = 1, but scattering imparts a frequency-dependent phase on the photon. The importance of the frequency-dependent phase is highlighted when scattering Gaussian pulses off the cavity–QED system. Defining ${\rm{e}}^{i{\phi }_{1}}={t}_{1}$, we then have ${\phi }_{1}={-i\ln ({t}_{1})}$. As in standard Gaussian pulse propagation^18^, the first to third derivatives of *ϕ*_1_ then give the delay Δ*τ*_1_(*Δ*_L_, *Δ*_C_), broadening and chirp, and distortion *d*_1_(*Δ*_L_, *Δ*_C_) of the Gaussian pulse upon scattering off the quantum system, respectively. On resonance (*Δ*_L_ = 0, *Δ*_C_ = 0), the delay is Δ*τ*_1_(0, 0) = 4/*Γ*, where *Γ* = 4*g*^2^/*κ* is the Purcell-enhanced decay rate. The distortion is given by *d*_1_(0, 0) = −32(1 − 3*Γ*/*κ*)/*Γ*^3^.

The physics of two-photon scattering is richer, as the energy of the individual photons is not necessarily conserved, which leads to photon correlations. The two-photon scattering matrix has previously been computed^16,19^, but here we diagonalise the scattering matrix and show that the two-photon eigenstates contain a subspace of two-photon bound states (photonic dimers) (the full calculation is provided in Supplementary Section VI). We find general semi-analytic forms for these states, but in the limit where *κ* is larger than all other rates and detunings in the system, the bound eigenstates have the simple form

2 $${{\psi }_{E}({x}_{\rm{c}},\,x)} = {N{\rm{e}}^{iE{x}_{\rm{c}}}\left[{\rm{e}}^{-\frac{{{\varGamma }}}{2{v}_{\rm{g}}}(1+\frac{{{\varGamma }}}{\kappa })| x| }-\frac{{{\varGamma }}}{\kappa }{\rm{e}}^{-\frac{\kappa }{2{v}_{\rm{g}}}| x| }\right]+O\left(\frac{1}{{\kappa }^{2}}\right)},$$

where *N* is a normalisation constant and *O*(1/*κ*^2^) indicates terms of order 1/*κ*^2^ and higher. The state is not separable, and the two photons forming the bound state are entangled with each other. In the relative two-photon coordinate *x* = *x*_1_ − *x*_2_, the photons are exponentially localised. However, because the two-photon energy is conserved, they take the form of a plane wave ${\rm{e}}^{iE{x}_{\rm{c}}}$ in the two-photon centre-of-mass coordinate *x*_c_ = (*x*_1_ + *x*_2_)/2, with a common two-photon frequency *E*. The exponential localisation in the relative coordinates evidences the strong correlation of the two photons in the bound state. In contrast to the waveguide QED bound states^6^, the presence of the cavity results in the second term, which removes the cusp such that the function is smooth at *x* = 0.

The strong localisation within a time 1/*Γ* in the difference coordinate means that the two photons in the bound state excite and stimulate the emission of the atom. This distinctly correlated interaction between the photons leads to this eigenstate having its own distinct transmission coefficient *t*_B_(*E*) and dispersion in comparison to the single-photon eigenstate, and therefore undergoes different delays, broadening and distortion. We compute the general form of *t*_B_(*E*) numerically, but, in the limit of a broadband cavity, the transmission coefficient of the two-photon bound states is

3 $${t}_{\rm{B}}{(E)} = {\frac{E(\kappa +2{{\varGamma }})-2i{{\varGamma }}(\kappa -{{\varGamma }}-{E}^{2}/{{\varGamma }})}{E(\kappa +2{{\varGamma }})+2i{{\varGamma }}(\kappa -{{\varGamma }}-{E}^{2}/{{\varGamma }})}+O\left(\frac{1}{{\kappa }^{2}}\right)}.$$

Similar to single-photon scattering, by taking respectively the first and third derivatives of ${\phi }_{\rm{B}}={-i\ln ({t}_{\rm{B}}(E))}$, we find that the delay of the two-photon bound state in the centre-of-mass coordinate is Δ*τ*_2_(0, 0) = 1/*Γ* + 3/*κ* and the distortion is *d*_2_(0, 0) = −(1 − 3*Γ*/*κ*)/(2*Γ*^3^). In comparison to the single-photon state, the bound state therefore undergoes both a reduced delay and a factor of 64 less distortion. The reduction in distortion was shown to be related to soliton propagation in waveguide QED^6^.

To fulfil experimentally the two criteria to study the photon-number-dependent scattering dynamics, namely unidirectional light propagation and a strong atom–photon interaction, we employ not a real atom but an artificial atom, a single QD. The QD is embedded in a Fabry–Pérot microcavity. The cavity suppresses the effects of phonons such that the QD mimics a two-level system precisely^20^. The epitaxially grown InAs QDs are part of a semiconductor heterostructure comprising an n–i–p diode and a GaAs/AlAs Bragg reflector—the ‘bottom mirror’. The ‘top mirror’ consists of a concave, dielectric Bragg mirror fabricated into a silica substrate. The reflectivity of the bottom mirror is substantially higher than that of the top mirror. With the aid of *xyz* nanopositioners, one can position a QD in the sample relative to the cavity mode and one can tune the resonance frequency of the cavity to that of the QD’s emission. Essential for the unidirectionality condition, the cavity should have only one port. In this system, undesired losses (losses via the bottom mirror, absorption and scattering losses^21^) account for *κ*_loss_/(2π) = 0.72 ± 0.07 GHz (ref. ^22^), while the total cavity linewidth is *κ*/(2π) = 20.1 ± 1.5 GHz, indicating that ~96% of the light is back-reflected via the one port of the microcavity, namely the top mirror. The QDs in this sample present a close-to-transform-limited linewidth *γ*/(2π) = 0.30 GHz. When QD and cavity are coupled, the Purcell-enhanced QD linewidth *Γ* = *F*_P_ × *γ* becomes *Γ*/(2π) = 4.24 GHz, where *F*_P_ = 14.1 is the Purcell factor. The lifetime of the emitter becomes *τ*_QD_ = 37.5 ps, and the QD–cavity coupling rate *g*/(2π) = 4.62 GHz. The strong atom–photon interaction and near-lossless operation result in a near-unitary probability of emission from the QD into the cavity mode, *β* = *F*_P_/(*F*_P_ + 1) = 0.93. Further experimental details are provided in Supplementary Sections I and II.

The direct observation of photon-number-dependent scattering dynamics is presented in Fig. 1b. A weak, coherent Gaussian pulse with temporal full-width at half-maximum (FWHM) of 135 ps—about twice the lifetime of the QD, *σΓ* = 2.2—is launched into the input of the optical system. Without any interaction with the cavity–QED system, it propagates through the optical system and arrives at the single-photon detectors at time *τ* = 0 (the pulse peak is represented by the grey dotted line). Upon resonant interaction with the broadband cavity, but in the absence of the quantum emitter, the Gaussian pulse undergoes a linear transmission, and is delayed by Δ*τ*_C_ = 29.2 ± 0.4 ps (the centre of the Gaussian pulse is represented by the black dotted line). This delay matches the predicted delay for a one-sided cavity Δ*τ*_C_ = 4/*κ* = 31.7 ± 2.4 ps (Supplementary Section IV). The delay imparted via the elastic scattering of a wavepacket by a resonator is often referred to as a Wigner delay^23^. In the presence of the QD, that is, when the QD is tuned into resonance with the cavity, we observe that the scattered *n*-photon pulse reveals an *n*-dependent ‘quantum Wigner delay’. We inspect the dynamics under full resonant conditions, *Δ*_L_ = *Δ*_C_ = 0, via the *n*th equal-time correlators. The single-photon scattering is given by a power measurement, *G*^(1)^(*τ*), presented as red dots in Fig. 1b, and shows how the output pulse is delayed relative to the input pulse, a result also previously observed for other quantum systems^24–26^. The scattered pulse is non-Gaussian in shape. This distortion causes the peak of the pulse to be delayed by a value different from Δ*τ*_1_ = 4/*Γ* (Supplementary Fig. 6a–c). The distortion arises from the fact that the spectral components of the pulse probe a sizeable fraction of the components making up the resonance of the cavity–QED system. The observed dynamics in *G*^(1)^(*τ*) are well captured by the theoretical model (red solid line). The scattering of two-photon states is examined via *G*^(2)^(*τ*, *τ*) (Fig. 1b, blue dots; theory, solid blue line). We find experimentally that both the delay and distortion in *G*^(2)^(*τ*, *τ*) are significantly reduced compared to those of *G*^(1)^(*τ*). The theory accounts for the measured *G*^(2)^(*τ*, *τ*) very convincingly. This constitutes a clear observation of two-photon bound states. We also interrogate the third-order equal-time correlator, *G*^(3)^(*τ*, *τ*, *τ*), given by green dots in Fig. 1b (the green solid line is a Gaussian fit). Both the peak delay and temporal width of the *G*^(3)^(*τ*, *τ*, *τ*) curve are reduced further, consistent with the observation of three-photon bound states.

The finite spectral width of the pulses probes the frequency- and photon-number-dependent phase imparted on different photon-number states. For a coherent input pulse we examine the delay experienced by an *n*-photon pulse by comparing the delay at the peak of the scattered pulse ${{\Delta }}{\tau }_{n}:={\max ({G}^{(n)}(\tau ,\,\tau ,\,...,\,\tau ))}$ (Fig. 1c). Because longer pulses undergo significantly reduced distortion, for *n* = 1, 2 we extract the peak delay from measurements with ten different pulse widths (1.3 ≤ *σΓ* ≤ 26.0). The one- and two-photon delays correspond well to the theoretical predictions (red and blue dotted lines, respectively). The single-photon wavepackets undergo a delay Δ*τ*_1_ = 144.02 ± 26.90 ps, and two-photon wavepackets undergo a reduced delay Δ*τ*_2_ = 66.45 ± 5.97 ps. The reduced delay is a consequence of stimulated emission: the first photon excites the atom, and the second photon stimulates the emission of the atom, thereby reducing the total time in which the photons interact with the atom. The three-photon delay is Δ*τ*_3_ = 45.51 ± 0.09 ps, a further reduction. This measurement of the quantum Wigner delay therefore unveils the existence of few-photon bound states. Key to success is the strong nonlinear and unidirectional scattering off the single quantum emitter.

We proceed to examine the autocorrelation functions of the scattered pulses. Figure 1d shows the two-photon autocorrelation map *G*^(2)^(*τ*_ch1_, *τ*_ch2_) of the weak Gaussian pulses scattered off the optical cavity, but in the absence of the quantum emitter. The linear response of the cavity displaces the two-dimensional Gaussian pulse shape by Δ*τ*_C_ along the equal-time-of-arrival line (*τ*_ch1_ = *τ*_ch2_) with respect to the Gaussian structure of the non-interacting pulse centred at (*τ*_ch1_, *τ*_ch2_) = (0, 0). As shown experimentally (theoretically) in Fig. 1e(f), when the pulse interacts with the QD–cavity system the correlated counts are drawn towards the diagonal of the *G*^(2)^-map (white dashed line), and can no longer be described by a linear transformation of the response to the bare cavity. We examine also the three-photon autocorrelation map *G*^(3)^(*τ*_ch1_, *τ*_ch2_, *τ*_ch3_) in Fig. 1g, where the volumetric isosurfaces at 0.05, 0.2, 0.5, 0.75 and 0.90 of the normalised counts are shown. The projections on the axes are the cut-through planes in each of the detection channels at time *τ* = 0. As in the *G*^(2)^ measurements, there is a strong peak along the diagonal revealing highly correlated three-photon states, as well as faint lateral lobes away from the diagonal^27^. Figure 1h displays a cut-through along the plane defined by *τ*_ch1_ = *τ*_ch2_ and *τ*_ch3_ where the clustering of the coincidence counts along the diagonal are prominently revealed. In all likelihood, this manifests the propagation of three-photon bound states (photonic trimers).

**Fig. 2:**
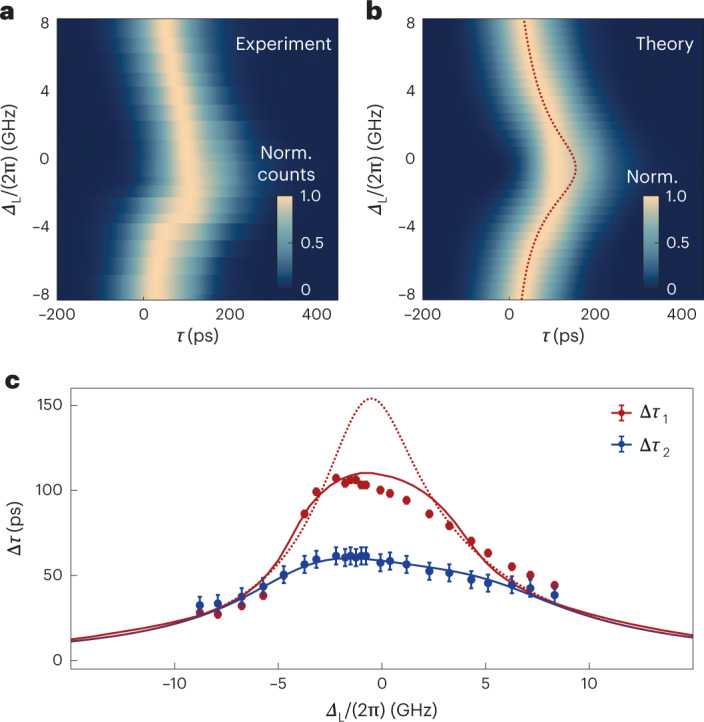
Single-photon and two-photon bound state delay dispersion. **a**,**b**, Experimental *G*^(1)^(*τ*) as a function of laser detuning *Δ*_L_/(2π) for a cavity–QD detuning of *Δ*_C_/(2π) = 1.0 GHz and for an input pulse with FWHM intensity of ~135 ps (**a**), and the respective simulation, where the red dotted line indicates the single-photon component delay in the continuous-wave limit (**b**). **c**, Peak delay Δ*τ* as a function of laser detuning for single photons (red) and two-photon bound states (blue). The solid lines are the numerically simulated peak delays for the single- and two-photon bound states. The red dotted line shows the calculated pulse delay Δ*τ*_1_ neglecting distortion. In this system, the two-photon bound state propagates without noticeable distortion. Error bars arise from fitting residual standard error. Source data

Next, we investigate the behaviour of the single- and two-photon scattering dynamics as a function of the central frequency of the photons. Figure 2a,b shows, respectively, the experimental and simulated power signal of the scattered pulse (FWHM = 135 ps) as a function of laser detuning from the QD’s resonance *Δ*_L_/(2π). The red dotted line in the theoretical model shows where the pulse maximum would occur if distortion effects were disregarded. Here, the cavity is slightly detuned from the QD, *Δ*_C_/(2π) = 1.0 GHz, which induces a slight spectral asymmetry to the one- and two-photon peak delays presented in Fig. 2c (red and blue dots, respectively). The results are in good agreement with the simulations and validate the theoretical model. We calculate numerically the dispersion of the peak delays Δ*τ*_1_ and Δ*τ*_2_ (red and blue solid lines). The results describe the experimental observations very well. Here too, the red dotted line corresponds to the single-photon case, neglecting pulse distortion. The results demonstrate that two-photon bound states experience a much reduced distortion, imperceptible in this system.

**Fig. 3:**
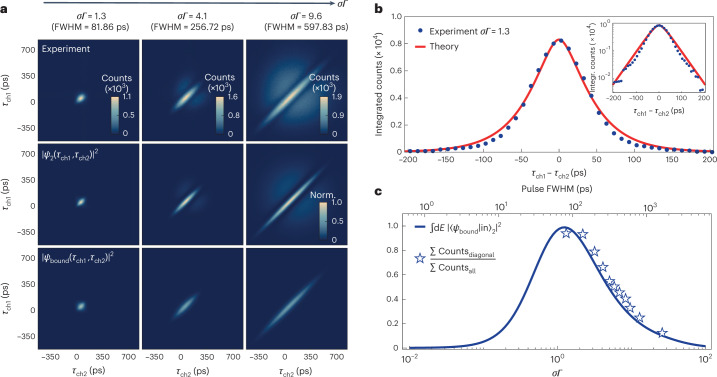
Observation of two-photon bound states as a function of input pulse width. **a**, Experimental (top row) and simulated (middle row) *G*^(2)^(*τ*_ch1_, *τ*_ch2_), as well as the two-photon bound-state contribution (bottom row) for increasing relative pulse widths *σΓ* = (1.3, 4.1, 9.6) (left to right). **b**, Integrated counts versus *τ*_ch1_ − *τ*_ch2_ for *σΓ* = 1.3 (blue dots) along with the theoretical result (red curve) for the two-photon bound states. Inset: the same, but on a log scale. Apart from the signal amplitude at *τ*_ch1_ − *τ*_ch2_ = 0, there are no fit parameters. **c**, Theory: calculated overlap of the two-photon bound state and an input state ${\left\vert {{{\rm{in}}}}\right\rangle}$, a two-photon Gaussian pulse of width *σ*. Experiment: integrated counts over the diagonal divided by the total integrated counts in each *G*^(2)^ map. The nodal line, clearly visible in the *G*^(2)^ map for *σΓ* = 9.6, is used to define the integration area for the diagonal; the same area is used for each *σΓ*. Both theory and experiment are plotted as a function of pulse width (intensity FWHM, top *x* axis) and relative pulse width normalised to the decay rate *Γ* (bottom *x* axis), which has units of (s s^−1^). Source data

The interaction of the two-photon wavefunction with the cavity–QED system strongly depends on the Gaussian pulse width. In Fig. 3a we explore this dependence, where we present on the top row the experimental *G*^(2)^ map for three different pulse widths, FWHM = (81.86, 256.72, 597.83) ps—equivalently *σΓ* = (1.3, 4.1, 9.6). We compare the experimental results to the simulated absolute square of the full two-photon wavefunction (middle row), which contains contributions from both two-photon bound states and extended states. The total contribution of the bound states alone is shown in the bottom row. The appearance of just the diagonals is evidence that the bound states contribute to the diagonal of the correlation maps. The extended states contribute to the lobes away from the diagonals. Details of the model are elucidated in Supplementary Section VI. The nodal line (absence of coincidence events) that occurs between the diagonal (the contribution from the bound states) and lobes (the contribution from the extended states) occurs due to the different phase the two states obtain after scattering. The phase approaches π for the bound state and is 0 for the extended states. For *σΓ* = 1.3, the lobes are very weak in both the experiment and accompanying theory: in Fig. 3b, we sum the counts in the *G*^(2)^ map over successive stripes parallel to the diagonal, that is, for successive values of *τ*_ch1_ − *τ*_ch2_. This procedure is equivalent to performing a conventional correlation measurement *g*^(2)^(*τ*_ch1_ − *τ*_ch2_) with continuous-wave excitation, except unnormalised. We find an exponential dependence of *G*^(2)^ on *τ*_ch1_ − *τ*_ch2_ (Fig. 3b, inset). This reveals, experimentally, the exponential decay of the two-photon bound-state wavefunction. The exponential dependence of the bound states as revealed in the experiments is well described by the theoretical model: we evaluate the absolute value squared of equation (2), taking the parameters established from the spectroscopy experiments, and find excellent agreement with the experiment (Fig. 3b, red solid line). Finally, the total fraction of the scattered wavefunction in the bound-state subspace depends on the overlap of the two-photon bound states with the two-photon input pulse. The theory shows that this overlap has a strong dependence on the input Gaussian pulse duration relative to the lifetime of the quantum emitter, and is largest and very close to unity when the input pulse has a duration ~1/*Γ*, as shown in Fig. 3c as a solid blue line. Experimentally, we estimate the bound-state fraction by evaluating the ratio of the counts in the diagonal to the total counts in each *G*^(2)^ map. The results (blue stars) follow the theoretical prediction convincingly.

We demonstrate here the ability to manipulate and identify highly correlated photonic states in time. The results reveal stimulated emission in its most canonical description, a single quantum emitter interacting with single photons^19^. This achievement represents an important landmark in the development of a variety of quantum technologies. Stimulated emission plays a central role, for instance in approximate quantum cloning of photons^28^, a key technology for quantum information processing and networking. The strong dependence of the propagated pulse on photon number can be enhanced by cascading such cavity–QED systems and enables a variety of important applications, such as photon sorting, photon-number-resolving detectors and Bell measurements^29–31^. The revealing of two-photon bound states upon interaction with a single atom is an appealing resource for the realisation of high-fidelity two-qubit photonic gates, such as controlled-phase gates^32^. Furthermore, the systematic generation of photonic dimers paves the way for substantial advances in quantum metrology^33^, and quantum-enhanced microscopy and lithography^34,35^.

## Online content

Any methods, additional references, Nature Portfolio reporting summaries, source data, extended data, supplementary information, acknowledgements, peer review information; details of author contributions and competing interests; and statements of data and code availability are available at 10.1038/s41567-023-01997-6.

## Supplementary information

Supplementary Information Supplementary Figs. 1–8 and sections I–VII.

## Data Availability

Source data are available for this paper at 10.5281/zenodo.7610568. All other data that support the plots within this Paper and other findings of this study are available from the corresponding authors upon reasonable request. Source data are provided with this paper.

## Source data

Source Data Fig. 1 Processed experimental data for g1, g2-diagonal and g3-diagonal and simulated data for g1 and g2-diagonal. Processed experimental data for g1, g2-diagonal and g3-diagonal.
Source Data Fig. 2 Processed experimental data for g1 and g2-diagonal, and simulated data for g1 (from real input pulse, and infinite pulse) and g2-diagonal.
Source Data Fig. 3 Processed experimental data from g2 maps, and fit. Processed experimental data from g2 maps, and data from numerical simulation.

## Code availability

The numerical simulation codes used in this work are available from S.M. upon reasonable request. The codes used to analyse raw experimental data in this work are available from N.T. upon reasonable request.
