# Supplementary material for: Photon bound state dynamics from a single artificial atom
Source: Nat Phys. 2023 Mar 20;19(6):857–62. doi: 10.1038/s41567-023-01997-6 (PMC10264240; doi:10.1038/s41567-023-01997-6)
Supplement: Supplementary file 1 — Supplementary Figs. 1–8 and sections I–VII. [file 41567_2023_1997_MOESM1_ESM.pdf]

nature physics

Article

https://doi.org/10.1038/s41567-023-01997-6

Photon bound state dynamics from a single artificial atom

In the format provided by the
authors and unedited

Supplementary Information: Photon bound state dynamics from a single artificial atom

Natasha Tomm, Sahand Mahmoodian, Nadia O. Antoniadis, Rüdiger Schott, Sascha R. Valentin, Andreas D. Wieck, Arne Ludwig, Alisa Javadi, and Richard J. Warburton

I. Microcavity and quantum dot characterisation

The quantum dot (QD) in a cavity system consists of two main elements. First, as shown in Supplementary Fig. 1a, we employ a semiconductor heterostructure consisting of an n-i-p diode with embedded self-assembled InGaAs quantum dots (QDs). This heterostructure is grown on top of a semiconductor distributed Bragg reflector (DBR) that acts as the planar "bottom" mirror of the cavity and is nearly 100% reflective – the total unwanted losses $\kappa_{\text{loss}}/(2\pi) \leq 0.72\,\text{GHz}$. Second, the "top" mirror consists of a fused-silica substrate into which an atomically-smooth crater with radius of curvature $R = 12\,\mu\text{m}$ is machined, onto which a dielectric DBR coating is deposited. Further details of the sample and the system are described in detail in Ref. [1], Supplementary Information.

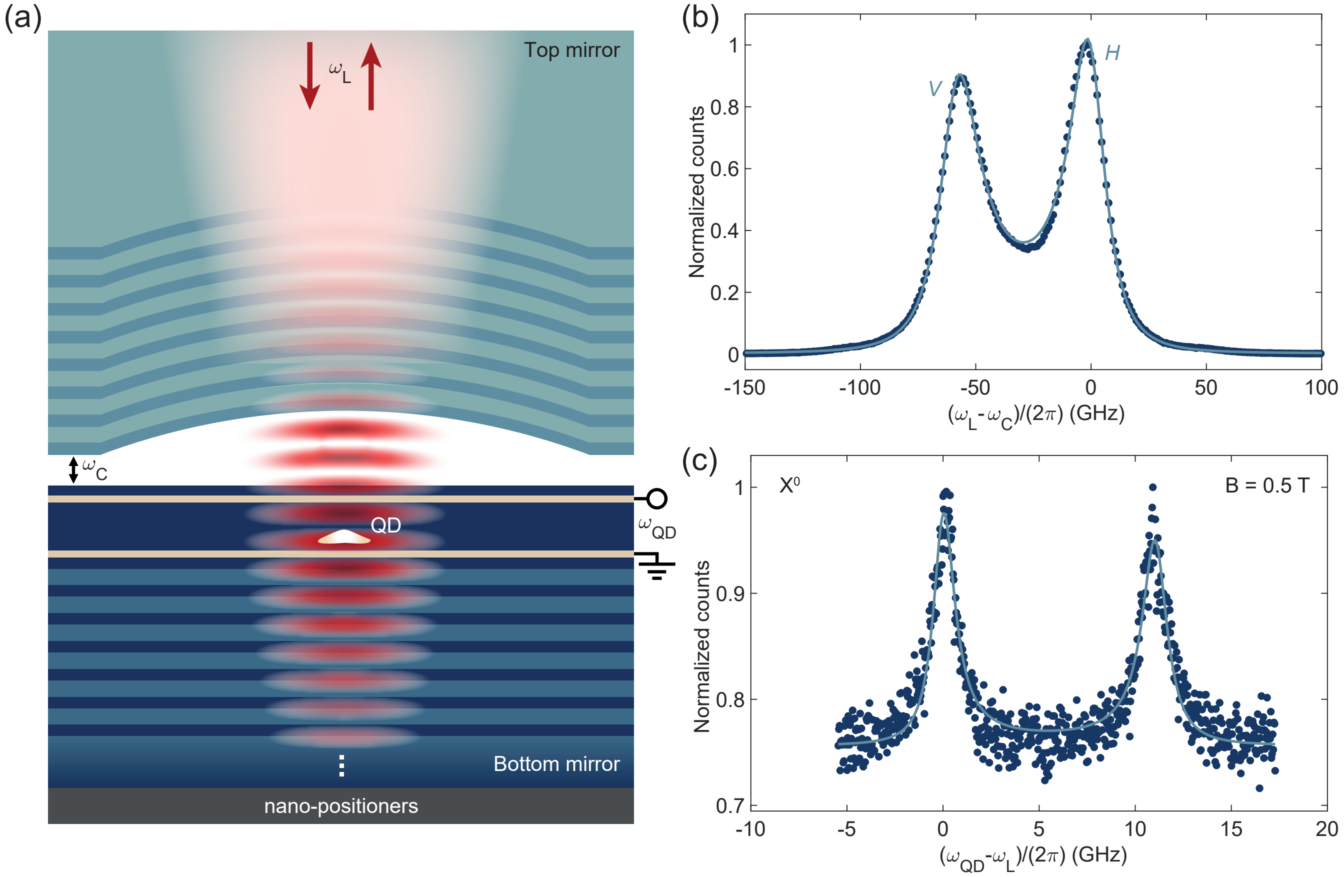

Supplementary Fig. 1. **Microcavity and quantum dot characterisation. (a)** Schematic (adapted from Ref. [1]) of the QD and one-sided cavity setup. The QDs are embedded in a diode structure which allows tuning angular frequency ω_{QD}. The cavity consists of separate top and bottom mirrors, which allows its angular frequency ω_{C} to be tuned with the help of nano-positioners. The laser light pulses with angular frequency ω_{L} are coupled into the cavity and exit via the top mirror. **(b)** Normalised signal versus laser detuning of the cavity resonance. The cavity resonance is split into two linearly- and orthogonally-polarised modes, H and V, separated by 58.8 GHz. The $\mathcal{Q}$-factor is measured to be $\mathcal{Q} = 16,200 \pm 1,200$, leading to a cavity loss rate $\kappa/(2\pi) = (20.1 \pm 1.5)\,\text{GHz}$. **(c)** Normalised signal versus QD detuning from the reference laser ($\lambda = 918.23\,\text{nm}$). The neutral exciton X^0 transitions in the QD are separated by a fine-structure splitting (FSS). Upon application of an out-of-plane magnetic field of B=0.5 T, the transitions move further apart, resulting in a FSS = $(11.0 \pm 0.1)\,\text{GHz}$. Application of the magnetic field allows us to work with just one of the QD transitions (the lower-frequency one).

The open-access nature of the cavity allows the tuning of its optical resonance (angular frequency ω_{C}) with the help of nano-positioners. One can also change the angular frequency of the input laser light ω_{L} which enters and exits the system. A cavity scan is shown in Supplementary Fig. 1b, where the splitting of the fundamental mode into two orthogonal H and V linearly-polarised modes can be observed [2], MS = $(\omega_{\mathrm{V}} - \omega_{\mathrm{H}})/(2\pi) = -58.8\,\mathrm{GHz}$. The mode-splitting is caused by residual strain in the semiconductor heterostructure. By fitting a double-Lorentzian line one extracts the total cavity loss rate $\kappa/(2\pi) = (20.1 \pm 1.5)\,\mathrm{GHz}$, equivalently a quality factor $\mathcal{Q} = 16,200 \pm 1,200$. Upon application of a voltage across the gates of the diode, the QD transition frequencies ω_{QD} can be tuned using the DC-Stark effect. We use the transitions of a neutral exciton X^0, which exhibits a so-called fine-structure splitting (FSS): there are two non-degenerate, linearly-polarised dipole moments. In order to work with one transition only, we search for a highly strained region in the sample in which both the FSS of the QD and the mode-splitting of the cavity are large. We select a QD with a starting linear frequency splitting FSS = $(7.6 \pm 0.1)\,\mathrm{GHz}$. We apply an out-of-plane magnetic field B=0.5 T, large enough to push the transitions further apart via the Zeeman effect, but not large enough to influence significantly the selection rules. (In a large magnetic field, the Zeeman splitting becomes much larger than the FSS such that the transitions become circularly polarised.) With this magnetic field strength, we finally have FSS = $(11.0 \pm 0.1)\,\mathrm{GHz}$ as shown in Supplementary Fig. 1c. We focus the rest of the discussion on the lower-frequency transition.

II. Coupled quantum dot-cavity system

We characterise the coupled QD-cavity system. We use input-output theory and follow the procedure described in Ref. [3, 4] to describe the interaction between the QD transition of interest, a two-level system (TLS), and two orthogonally and linearly polarised cavity modes. We examine the case of a continuous-wave, low-power input laser field of amplitude b_{in} with arbitrary polarisation,

$$b_{\mathrm{in}} = \alpha_H b_{\mathrm{in}} \mathbf{h} + \alpha_V b_{\mathrm{in}} \mathbf{v}, \tag{1}$$

where $\alpha_{H/V}$ are the complex coefficients of the Jones vectors in the orthonormal basis $\mathbf{h} = (1\ \ 0)^{\intercal}$ and $\mathbf{v} = (0\ \ 1)^{\intercal}$.

We consider a one-sided cavity with two optical modes along $\mathbf{h}$ and $\mathbf{v}$, both coupled to the continuum of modes with decay rate $\kappa_H = \kappa_V = \kappa$. We use the rotating frame of the quantum emitter. The field reflected by the QD-cavity system b_{r} is

$$b_{\mathrm{r}} = \epsilon_H b_{\mathrm{r}}^H \mathbf{h} + \epsilon_V b_{\mathrm{r}}^V \mathbf{v}, \tag{2}$$

with the probed reflected field amplitude in each polarisation given by

$$b_{\mathrm{r}}^{H/V} = \alpha_{H/V}\, b_{\mathrm{in}} \left(1 - t_{H/V}\right) - i\, t_{H/V}\, \Gamma_{H/V}\, \sigma_-, \tag{3}$$

where σ_- is the expectation value of the Pauli lowering operator for the TLS in the low-power regime. We denote $\Gamma_{H/V} = 4g_{H/V}^2/\kappa$ as the Purcell-enhanced atomic decay rate due to the interaction, with $g_{H/V}$ the coupling constant between the linear dipole moment of the TLS and each cavity mode. We note that for linear transitions, $g_{H/V}$ is real and given by the projection of the TLS's linear dipole moment onto the cavity mode's linearly-polarised vacuum electric field. We introduce also the transmission through the cavity modes in the absence of the quantum emitter:

$$t_{H/V} = \frac{1}{1 + 2i\left(\frac{\Delta_{H/V} - \Delta_{\mathrm{L}}}{\kappa}\right)}, \tag{4}$$

where $\Delta_{H/V} = \omega_{\mathrm{C}}^{H/V} - \omega_{\mathrm{QD}}$ is the detuning of the H/V polarised cavity mode with respect to the QD transition, and $\Delta_{\mathrm{L}} = \omega_{\mathrm{L}} - \omega_{\mathrm{QD}}$ is the laser detuning with respect to the QD transition. The lowering operator σ_- is responsible for "coupling" the two polarised optical modes and described as

$$\sigma_- = \frac{i\, t_H \sqrt{\Gamma_H}\, \alpha_H\, b_{\mathrm{in}} + i\, t_V \sqrt{\Gamma_V}\, \alpha_V\, b_{\mathrm{in}}}{t_H \frac{\Gamma_H}{2} + t_V \frac{\Gamma_V}{2} - i\, \Delta_{\mathrm{L}} + \frac{\gamma}{2}}, \tag{5}$$

where we account for the atomic decay rate into non-cavity optical modes via γ.

Finally, the scattered field amplitude is given by the ratio of reflected field and input field, namely

$$t_k = \frac{b_{\rm r}}{b_{\rm in}} = \epsilon_H \left[\alpha_H \left(1 - 2\,t_H + \frac{2\,t_H^2\,\Gamma_H}{t_H\,\Gamma_H + t_V\,\Gamma_V - 2i\,\Delta_{\rm L} + \gamma}\right) + \alpha_V \left(\frac{2\,t_H\,\sqrt{\Gamma_H}\,t_V\,\sqrt{\Gamma_V}}{t_H\,\Gamma_H + t_V\,\Gamma_V - 2i\,\Delta_{\rm L} + \gamma}\right)\right] + \epsilon_V \left[\alpha_H \left(\frac{2\,t_V\,\sqrt{\Gamma_V}\,t_H\,\sqrt{\Gamma_V}}{t_V\,\Gamma_V + t_H\,\Gamma_H - 2i\,\Delta_{\rm L} + \gamma}\right) + \alpha_V \left(1 - 2\,t_V + \frac{2\,t_V^2\,\Gamma_V}{t_V\,\Gamma_V + t_H\,\Gamma_H - 2i\,\Delta_{\rm L} + \gamma}\right)\right]. \quad (6)$$

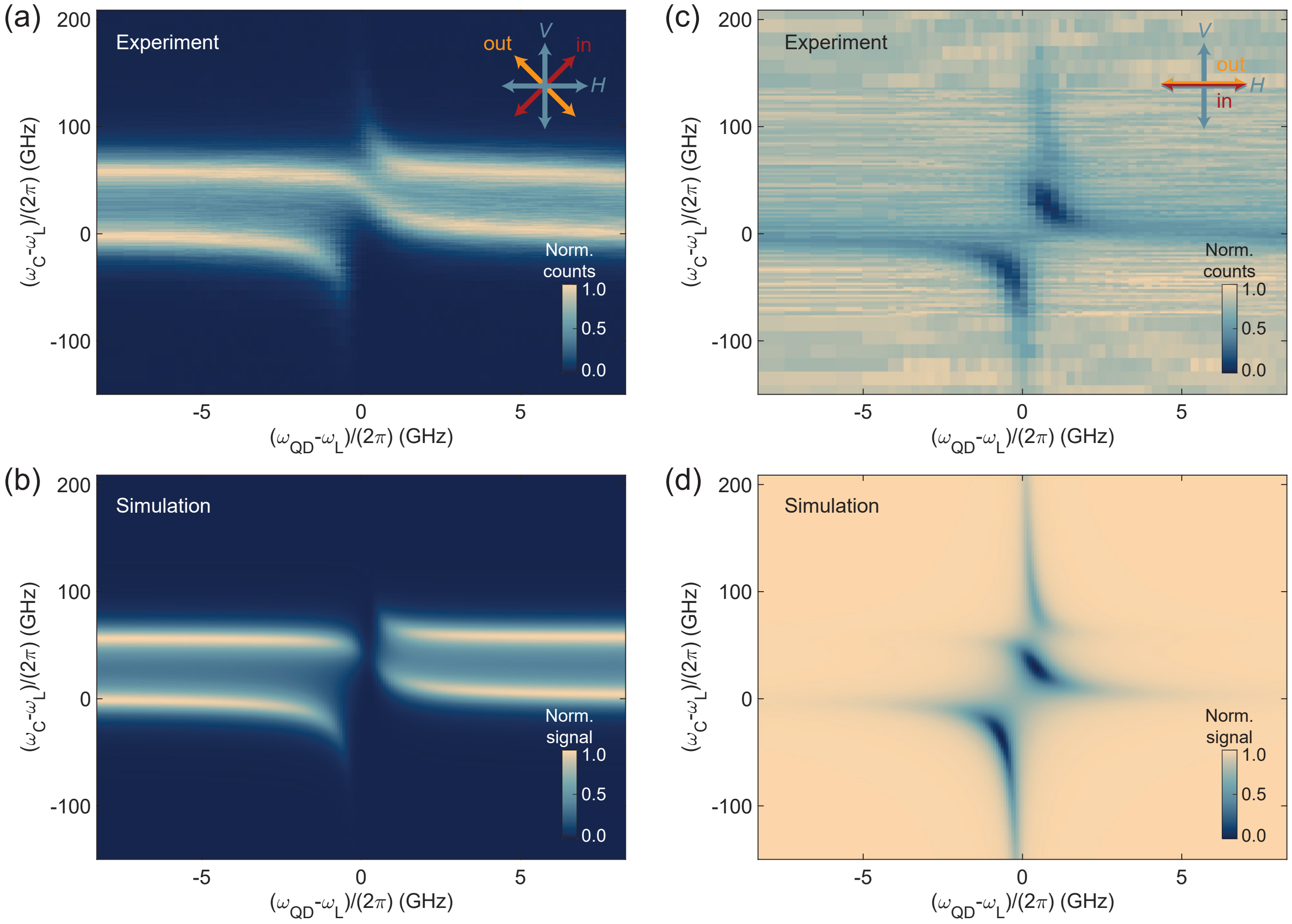

Supplementary Fig. 2. Resonant response of a QD in a one-side microcavity. (a) Transmission of laser light through the coupled QD-cavity system as a function of detuning from the cavity and the QD resonances. The measurement was performed in a cross-polarised mode (inset), where the input light is diagonal to the H and V cavity modes and the output polarisation is anti-diagonal. By fitting a Lorentzian function to the cut-through lines at the resonances of the two cavity modes, one can extract the Purcell-enhanced decay rate of the QD, $\Gamma_H/(2\pi) = 4.24\,$GHz and $\Gamma_V/(2\pi) = 1.30$. **(b)** Simulation of the transmission through the QD-cavity system using the parameters extracted from the experiment. **(c)** Spectrum of the back-reflected light from the QD-cavity system. The measurement was performed in co-polarised mode (inset), where input and output modes are horizontally polarised, aligned to the H polarised cavity mode, and **(d)** respective simulation.

Experimentally, we operate the system with the aid of a cross-polarised microscope [1, 5], and we probe the reflected light intensity $|t_k|^2$. We first study the properties of the TLS-cavity interaction in what we call "transmission" mode, shown in Supplementary Fig. 2a. Even though the signal is effectively reflected by the system, mathematically it is equivalent to probing the signal transmitted through a two-sided cavity with two orthogonal cavity modes. We achieve this by sending in linear-polarised light "diagonal" to the cavity modes, and collecting light on the anti-diagonal, as depicted in the inset of Supplementary Fig. 2a. In Eq. 6 this means that the input coefficients are $\alpha_H = 1/\sqrt{2}$, $\alpha_V = 1/\sqrt{2}$, the output coefficients $\epsilon_H = 1/\sqrt{2}$, $\epsilon_V = -1/\sqrt{2}$. We extract the Purcell-enhanced decay rate into each mode by taking the full-width-at-half-maximum (FWHM) of the two cavity resonances, $\Gamma_H/(2\pi) = 4.24\,$GHz and $\Gamma_V/(2\pi) = 1.30\,$GHz. This implies respective coupling constants $g_H/(2\pi) = 4.62\,$GHz and $g_V/(2\pi) = 2.55\,$GHz. The

non-cavity losses are given by the natural decay rate of the emitter, $\gamma/(2\pi) = 0.30\,\text{GHz}$, a parameter known from previous experiments [1, 6]. We estimate the Purcell factors $F_P^H = 14.1$ and $F_P^V = 4.3$. We simulate the response of the system with the parameters determined experimentally and using Eq. 6. The result is shown in Supplementary Fig. 2b. We note that the QD transitions are not perfectly linearly polarised due to the Zeeman effect which induces a slight ellipticity to the transitions.

Next, we examine the system in what we call "back-reflection" mode, in which both input and output fields are aligned to one of the linearly polarised cavity modes. In this work, we focus on the H cavity; it interacts more strongly with the lower energy transition of the QD. In this case, for the input and output polarisation coefficients, we have $\alpha_H = 1$, $\alpha_V = 0$ and $\epsilon_H = 1$, $\epsilon_V = 0$. The experimental and simulated results are presented in Supplementary Fig. 2c,d. We remark that in the case of a TLS with decay rate Γ coupled to a single cavity mode with coupling rate g and $\gamma = 0$, the amplitude reflection coefficient would be unity across the entire spectrum. In this limit the β-factor, defined as $\beta = F_P/(F_P + 1)$, is unity and the amplitude reflection coefficient reduces to Eq. 1 of the main text. At resonance the amplitude reflection coefficient is -1, which indicates that the light is scattered with unity intensity but undergoes a π phase shift, a useful condition for photon-photon quantum gates [7]. This limit is taken in the theoretical modelling throughout this work, see Sec. VI.

In this work, the unity β-factor condition is almost achieved. We take advantage of the tunable nature of the miniaturised Fabry-Perot cavity to maximise the β-factor. Employing xy-nanopositioners one can position the QD relative to the optical axis of the cavity, and in this way tune the β-factor, and respectively the QD's decay rate/lifetime. In Supplementary Fig. 3, we show the extracted $\Gamma/(2\pi)$ (blue dots, left axis) and equivalent emitter lifetime $\tau_{\text{QD}} = 1/\Gamma$ (red dots, right axis) as a function of the calculated β for different lateral positions of the QD relative to the cavity's optical axis. Here, β is calculated from the derived coupling constant $g = g_H$ at each position, and the known κ and γ. For all experiments in this manuscript we work in the condition of maximised coupling, with parameters $\beta = 0.934$, decay rate $\Gamma/(2\pi) = 4.24\,\text{GHz}$ and respective lifetime $\tau_{\text{QD}} = 37.5\,\text{ps}$. The average photon number per lifetime needed to saturate the quantum emitter in a one-sided cavity [3, 4], i.e. the "critical photon number" $\bar{n}_c$, is given by $\bar{n}_c = 1/(8\beta) = 0.1338$.

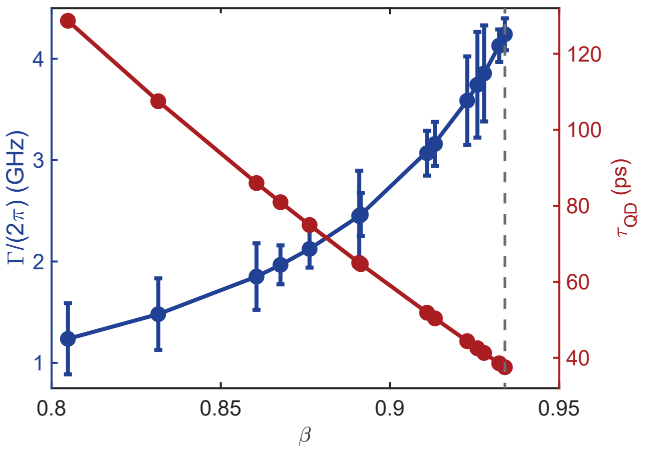

Supplementary Fig. 3. Tuning the lifetime of the QD coupled to the microcavity. The open-access microcavity enables tuning the lateral position of the QD relative to the centre of the cavity field, modifying *in-situ* the coupling rate g, respectively the β-factor. We determine experimentally the Purcell-enhanced linewidth of the better-coupled dipole transition $\Gamma = \Gamma_{\text{H}}$ (left axis, blue data points) and its respective expected lifetime τ_{QD} (right axis, red data points). When the QD is well centred with the cavity mode (dashed gray line), $\beta = 0.934$, $\Gamma/(2\pi) = 4.24\,\text{GHz}$ and $\tau_{\text{QD}} = 37.5\,\text{ps}$. Error bars for Γ are fitting residual standard error.

III. Experimental setup

The experimental setup for the generation and detection of single photons and photonic bound states consists of five segments, as shown in Supplementary Fig. 4. We employ a tunable continuous-wave laser (TOPTICA Photonics A.G., Germany) and select the laser power sent to the system with an acousto-optic modulator (AOM) controlled digitally by a PID-loop. We create electric Gaussian-shaped signals with FWHM between 75 ps and 2 ns with an arbitrary wave generator (AWG). A trigger signal is sent to a time-correlator to synchronize the experiment, and the analog output from the AWG is used to modulate an electro-optic modulator (EOM). The generated light pulses are sent into a 99:1

fibre beam-splitter, which we employ as an approximate optical circulator. We send 1% of the light intensity into the QD-cavity system, which resides in a helium bath-cryostat at a temperature of 4.2 K. The light is back-scattered and 99% of it is sent to a 50:50 fibre-beam splitter and finally to two superconducting nanowire single-photon detectors (SNSPDs, Single Quantum B.V., The Netherlands). The time-tags of the single-photon detection events are recorded with a correlator (Time Tagger, Swabian Instruments, Germany). In the experimental determination of a three-photon correlation function $G^{(3)}$, we divide the signal once more with a second 50:50 fibre-beam splitter and use four SNSPD detectors.

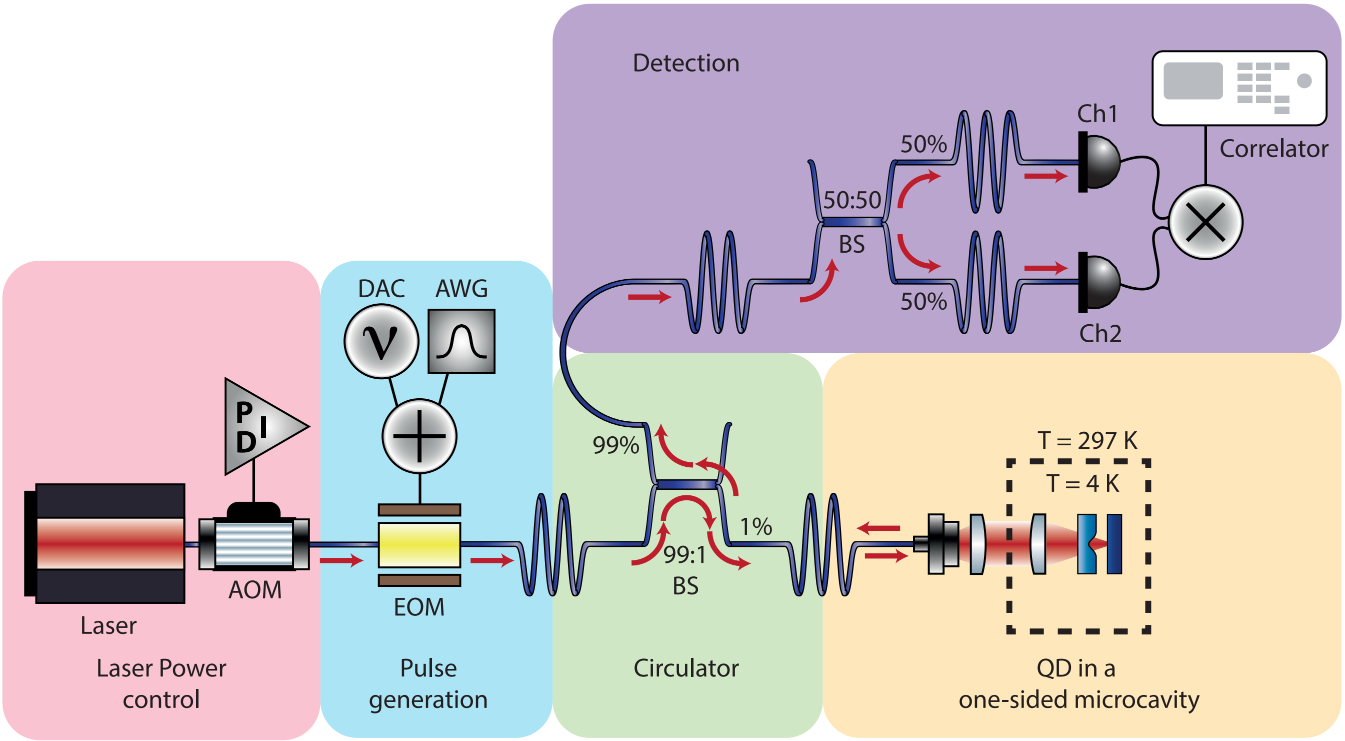

Supplementary Fig. 4. **The experimental setup.** Overview of the experimental setup for the measurement of the single photon and two-photon scattering dynamics. Light from a CW laser passes through an AOM (acousto-optic modulator) controlled via a PID loop in order to control the laser power. Laser light is sent to an EOM (electro-optic modulator) in order to create Gaussian pulses. These pulses are sent to the QD in a one-sided microcavity via a mock-circulator composed of a 99:1 fibre BS (beam-splitter). In the circulator, 1% of the input light is sent to the QD-cavity system, but 99% of the back-reflected light (co-polarised) is sent to the detection setup consisting of a Hanbury Brown-Twiss setup (a 50:50 BS with two detectors, Ch1 and Ch2, and a correlator).

IV. Wigner delay induced by a one-sided cavity

The elastic scattering of a wavepacket by a resonator is not an instantaneous process. It was shown by E.P. Wigner in 1955 that the scattered wave propagates with a time delay with respect to an unscattered wave [8]. The so-called "Wigner delay" is given by the derivative of the phase shift acquired by the wave with respect to its frequency [9]. The delay is frequency dependent. In the case of a one-sided optical cavity with an inverse lifetime κ the Wigner delay inherited by the interacting pulse is given by

$$\Delta\tau_C(\omega_L, \omega_C) = \frac{4}{\kappa} \frac{1}{1 + 4\left(\frac{\omega_\mathrm{L} - \omega_\mathrm{C}}{\kappa}\right)^2}. \tag{7}$$

It is easy to see that at resonance ($\omega_\mathrm{L} = \omega_\mathrm{C}$) the Wigner delay reduces to $\Delta\tau_C(0,0) = 4/\kappa$. For our cavity, the expected Wigner delay at resonance is $\Delta\tau_\mathrm{C}(0,0) = 4/\kappa = (31.7 \pm 2.4)$ ps.

We study the dynamical response of a $\sim$135 ps Gaussian pulse scattered by the one-sided cavity. We work in "back-reflection" mode, such that the input light only interacts with the H cavity mode. Supplementary Fig. 5a shows the

time of arrival of the pulse $G^{(1)}(\tau)$ as a function of detuning between the laser and the cavity resonance. Even for the shortest pulses used here, the scattered pulse is shape-maintaining over the entire spectrum. We evaluate the delay of the pulse peak $\Delta\tau$ for one- (red dots) and two-photon components (blue dots) as a function of detuning (Supplementary Fig. 5b). The delay experienced in both cases is the same, and described by Eq. 7, which we fit to the data (red and blue solid lines) allowing us to retrieve the cavity linewidth $\kappa_{\rm fit}/(2\pi) = (21.6 \pm 0.2)$ GHZ. This value is the same (within the error bars) as the value of κ determined via the intensity measurement described in Section I, $\kappa/(2\pi) = (20.1 \pm 1.5)$. The photon lifetime in the cavity is $\tau_{\rm C} = 1/\kappa_{\rm fit} = (7.3 \pm 0.1)$ ps, leading to a Wigner delay at resonance of $\Delta\tau_{\rm C}(0,0) = 4/\kappa_{\rm fit} = (29.2 \pm 0.4)$ ps. Finally, we show in Supplementary Fig. 5c that the Wigner delay for a classical resonator is linear, i.e. independent of input laser power. For the experiments in the main manuscript we use the average of the delays extracted in this measurement as the "classical" (i.e. cavity-only) baseline.

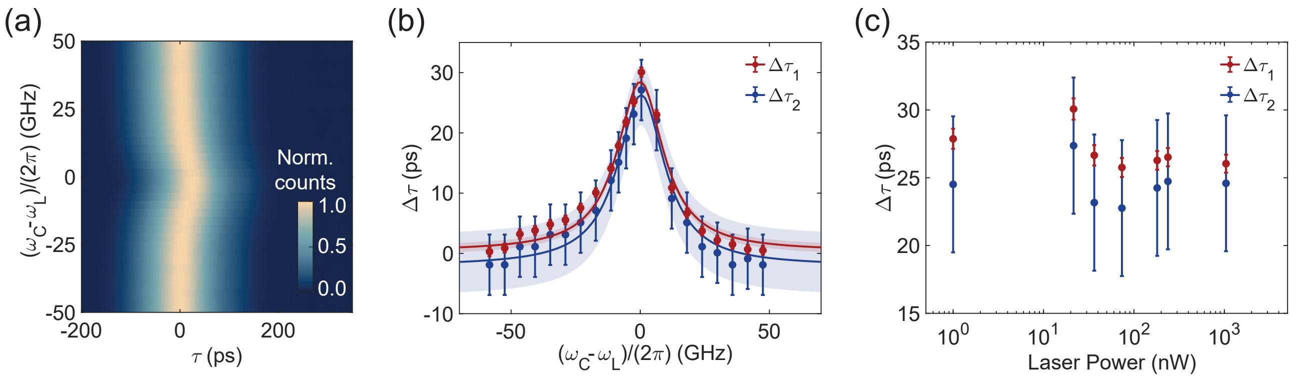

Supplementary Fig. 5. **Wigner delay induced by a one-sided cavity. (a)** Measured $G^{(1)}(\tau)$ histogram as a function of cavity detuning. **(b)** Measured pulse peak delay $\Delta\tau$ of $G^{(1)}$ (red data points) and $G^{(2)}$ (blue data points) as a function of cavity detuning. For an optical cavity (a classical resonator), the Wigner delay is photon-number independent, and at resonance equal to $\Delta\tau_C = 4/\kappa$ for a one-sided cavity. By fitting Eq. 7 (solid lines) we determine $\kappa_{\rm fit}/(2\pi) = (21.6 \pm 0.2)$ GHZ. Error bars in data points arise from fitting residual standard error, and error bars in fit arise from accounting for experimental error bars in the fitting process. **(c)** The Wigner delay for single-photon states (red data points) and two-photon states (blue data points) at resonance measured for different laser powers: the delay is independent of the power. Error bars in data points arise from residual standard error.

V. Properties of the photon-number dependent scattering

The scattered light dynamics depend on the input pulse shape and temporal width. Shorter pulses in time, a few times the lifetime of the emitter, undergo higher-order distortion as more spectral components interact with the TLS (see Sec. VI). Much longer pulses are spectrally narrow and the continuous-wave (CW) limit is appropriate in which only a delay (phase shift) is impinged onto the pulse. In Supplementary Fig. 6a, the normalised power $G^{(1)}(\tau)$ as a function of QD–laser detuning ($\Delta_{\rm QD} = \omega_{\rm QD} - \omega_{\rm L}$) is shown. The laser is in resonance with the cavity. We show the one-photon scattering response for two pulse widths, a shorter one (left, pulse intensity-FWHM of 81.86 ps, corresponding to $\sigma\Gamma = 1.3$) and a longer one (right, pulse intensity-FWHM = 597.83 ps, corresponding to $\sigma\Gamma = 9.6$). In both cases, far from resonance, the pulse is delayed by the classical delay of the cavity alone and does not present any reshaping. In the shorter-pulse limit, the transmitted pulse is distorted near the resonance and no longer corresponds to a Gaussian pulse, as is seen in Supplementary Fig. 6b. The distortion of the pulse results in a deviation of the peak delay $\Delta\tau$ in shorter pulses compared to the infinite-pulse limit, in which the distortion is negligible. The two-photon auto-correlation maps for these pulse widths are discussed in the main text. We probe the peak delay at resonance for single photons $\Delta\tau_1$ and for two-photon states $\Delta\tau_2$ as a function of pulse FWHM, displayed in Supplementary Fig. 6c, where the red and blue dashed lines correspond to the theoretical prediction neglecting the effects of distortion. We point out that the single-photon scattering dynamics are more sensitive to dispersion effects (see Fig. 2 of main text) than the dynamics of the two-photon bound states; this explains the larger variance in $\Delta\tau_1$ in this analysis.

The response of a single TLS is highly susceptible to the number of photons impinging on it, as the TLS saturates upon absorption of a single photon [3, 10]. This results in a strong nonlinear response even at very low input laser powers. We expect the few-photon nonlinearity to fade with strong coherent fields, as in this regime a large fraction of the pulse is scattered without interacting with the saturated TLS. Supplementary Fig. 6d presents the normalized power-time response as a function of QD-detuning from the laser-cavity resonance for a pulse with ∼135 ps FWHM

in the case of a low-power (2.89 nW, left) and high-power (73.64 nW, right) input. These laser powers (measured at the "power control" setup) translate to an average photon-number per lifetime of $\bar{n} = 0.0052$ and $\bar{n} = 0.1313$ at the cavity, respectively. The QD saturates at the critical photon number [4] $\bar{n}_c = 1/(8\beta) = 0.1338$. In the latter measurement, the reduced delay at resonance testifies to the saturation of the QD. The fingerprints of two-photon correlated states (clustering and delay of coincidence counts along the diagonal) also diminish (Supplementary Fig. 6e). We present in Supplementary Fig. 6f the peak delay observed for one (red) and two photons (blue) as a function of average photon number per lifetime. At low photon-number, the measurements correspond well to the simulated $\Delta\tau$

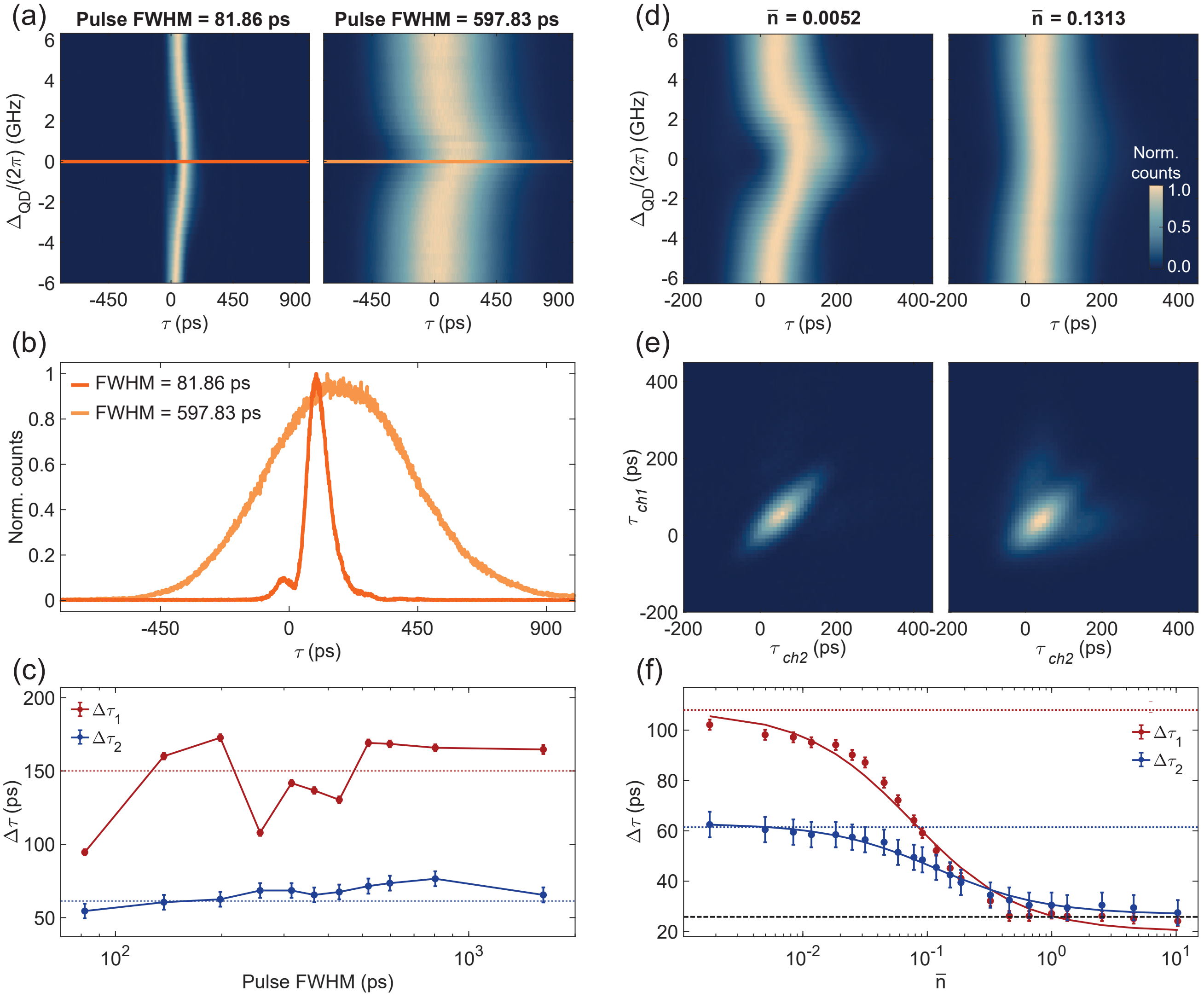

Supplementary Fig. 6. Input pulse width and input-power dependent dynamics. (a) Time of arrival τ of the propagated pulse power $G^{(1)}(\tau_{\text{ch1}})$ as a function of QD detuning from the laser resonance $\Delta_{\text{QD}}/(2\pi)$ for a short (pulse FWHM = 81.86 ps, left) and a long (pulse FWHM = 597.83 ps, right) input Gaussian pulse. (b) Line cut-through of (a) at resonance, showing the pulse deformation for short pulses. (c) Peak delay $\Delta\tau$ as a function of input pulse width for one (red) and two photons (blue). The red and blue horizontal dashed lines represent the theoretical values in the infinite-pulse-width limit. (d) Time of arrival τ of the propagated pulse power $G^{(1)}(\tau_{\text{ch1}})$ for a 135 ps Gaussian pulse as a function of QD detuning from the laser resonance for an average input photon number $\bar{n} = 0.0052$ (left) and $\bar{n} = 0.1313$ (right), and (e) respective $G^{(2)}(\tau_{\text{ch1}}, \tau_{\text{ch2}})$ maps at resonance. (f) Peak delay as a function of average input photon number for one- (red) and two-photon (blue) states. Solid lines are fits to the TLS-saturation relation, with critical photon number $\bar{n}_c = 0.1338$. The red and blue dashed lines show the modelled $\Delta\tau$ for one- and two-photon states for a 135 ps Gaussian pulse in the low power limit, and the black dashed line is the delay of the cavity alone. Data point centres and error bars in (c),(f) are the maximum of fitted Gaussian distribution and residual standard errors from fitting, respectively.

for this pulse width (red and blue dashed lines), converging gradually as the laser power increases to the limit where only the classical delay, induced by the cavity alone, remains (black dashed line). The delay response for both one- and two-photon states correspond well to a TLS-saturation power law like function,

$$\Delta\tau(\bar{n}) = 1 - (1 - \Delta\tau^{\infty}/\Delta\tau^{0}) \cdot \frac{\bar{n}/\bar{n}_c}{1 + \bar{n}/\bar{n}_c}, \tag{8}$$

where $\Delta\tau^0$ and $\Delta\tau^{\infty}$ are the peak delays measured in the limits of zero and infinite input photons, respectively. The fits are depicted with red and blue solid lines in the plot.

Next, we evaluate $G^{(1)}(\tau)$ and the diagonals of the auto-correlation functions $G^{(2)}(\tau,\tau)$ and $G^{(3)}(\tau,\tau,\tau)$ and determine the dynamical properties of the photon-number dependent scattering. We present in Supplementary Fig. 7a,b the values of peak delay $\Delta\tau$ and relative pulse width $\sigma_n/\sigma_{|in\rangle}$ as a function of photon-number n. For each n, these values are extracted from $G^{(1)}(\tau)$, $G^{(2)}(\tau,\tau)$ and $G^{(3)}(\tau,\tau,\tau)$. Here, we extract the average and variance from all experiments presented in the course of this work under low-power resonant conditions.

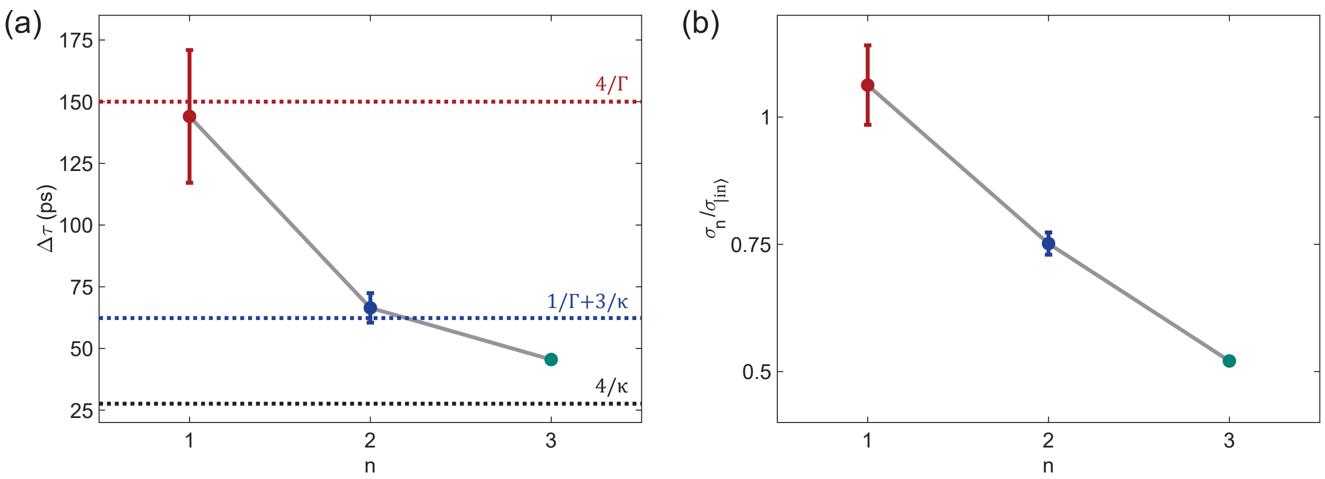

Supplementary Fig. 7. Photon-number-dependent scattering dynamics. (a) Average peak delay of the scattered pulse $\Delta\tau$, and **(b)** Average ratio of the measured pulse width after scattering (σ_n) to the input pulse width $\sigma_{|in\rangle}$, for different photon-numbers n. The values are the mean and 1-sigma standard deviation extracted from twelve (for n=1,2) and one (for n=3) low-power resonant experiments carried out in the course of this work (including differing pulse widths), probed via $G^{(1)}$, $G^{(2)}(\tau,\tau)$ and $G^{(3)}(\tau,\tau,\tau)$.

The single-photon components undergo a delay $\Delta\tau_1 = (144.02 \pm 27.90)$ ps, in good agreement with the predicted value of $\Delta\tau_1^{\text{theory}} = 4/\Gamma = 150.00$ ps. The large error stems from the sensitivity of the peak delay to distortions, as discussed in the main text and Section VI; the distortions depend sensitively on the exact detunings which can drift slightly. We determine the two- and three-photon delays to be $\Delta\tau_2 = (66.48 \pm 5.97)$ ps and $\Delta\tau_3 = (45.51 \pm 0.09)$ ps. The two-photon delay corresponds well to the prediction of $\Delta\tau_2^{\text{theory}} = 1/\Gamma + 3/\kappa = 61.3$ ps. We note that as we approach the classical limit of $n \to \infty$, the delay converges to the value induced by the cavity alone (see Section IV). Finally, we compare the Gaussian width of the interacting n-photon output pulse and the input pulse and determine $\sigma_1/\sigma_{|in\rangle} = 1.06 \pm 0.08$, $\sigma_2/\sigma_{|in\rangle} = 0.75 \pm 0.02$ and $\sigma_3/\sigma_{|in\rangle} = 0.52 \pm 0.00$.

VI. Theory

We provide a detailed derivation of the equations used to compute the results in the main text. Here we provide a scheme to compute the output state when considering a weak coherent input pulse with average photon number $\bar{n} \ll 1$. We use one- and two-photon scattering matrices that have previously been computed [11, 12]. In addition to this we compute the one- and two-photon delay and dispersion a pulse undergoes when interacting with a single-sided cavity QED system. In particular we do this for the one-photon pulse and we do this in the two-excitation center-of-mass coordinate for the two-photon bound state, whose form we also compute.

The TLS-cavity system can be described by the Jaynes-Cummings interaction, where the cavity is unidirectionally

coupled to a one-dimensional continuum of modes, specified by the Hamiltonian

$$\hat{\mathcal{H}} = -i\, v_g \int dx\, \hat{a}^\dagger(x)\, \partial_x \hat{a}(x) + \Delta_C\, \hat{c}^\dagger \hat{c} + \eta\, \left(\hat{a}(0)\hat{c}^\dagger + \hat{c}\hat{a}^\dagger(0)\right) + g\, \left(\hat{c}\hat{\sigma}_+ + \hat{\sigma}_- \hat{c}^\dagger\right). \tag{9}$$

The first two terms describe respectively the free-evolution of the waveguide mode at position x with annihilation operator $\hat{a}$ and the cavity field with annihilation operator $\hat{c}$ and cavity-atom detuning $\Delta_C = \omega_C - \omega_0$. Here, the energy has been renormalised to that of the atom and the dispersion of the mode continuum has been linearised about this point, and v_g is its group velocity. The last two terms express the waveguide-cavity exchange with coupling constant $\eta = \sqrt{\kappa v_g}$, where κ is the cavity decay rate, and the cavity-TLS interaction with coupling rate g. Here $\hat{\sigma}_-$ ($\hat{\sigma}_+$) is the Pauli lowering (raising) operator

A. The input state

We truncate the input coherent pulse with real-valued amplitude $\alpha = \sqrt{\bar{n}}$ at two photons,

$$|\text{in}\rangle \sim e^{-\alpha^2/2}\left[1 + \alpha \hat{a}^\dagger_{\text{in}} + \frac{\alpha^2}{2}\hat{a}^\dagger_{\text{in}}\hat{a}^\dagger_{\text{in}}\right]|0\rangle. \tag{10}$$

Here,

$$\hat{a}^\dagger_{\text{in}} = \int dx \hat{a}^\dagger(x) f(x) \tag{11}$$

creates a photon in the input mode with normalized spatial profile $f(x)$ (with equivalent annihilation operators). The input mode profile produced by the laser is approximately Gaussian. For an ideal Gaussian pulse with width σ and central frequency detuning from the atomic resonance $v_g k_0$ we have

$$f(x) = \frac{1}{\sqrt{\sigma}\pi^{1/4}} e^{ik_0 x} e^{-\frac{x^2}{2\sigma^2}}. \tag{12}$$

Here, v_g is the group velocity of the mode that is coupled to the cavity (see Fig. 1 of the main text for a schematic of the setup). In our experimental setup we have a free space mode with $v_g = c$.

B. Scattering Matrix

To compute the output state we apply photon scattering matrices on the one- and two-photon components of the coherent state. The one- and two-photon scattering matrices for a single-sided cavity QED setup have previously been reported [11, 12]. The single-photon scattering matrix can be written as a transmission coefficient in k-space such that scattering a photon maps

$$\hat{a}^\dagger(k) \to t_k \hat{a}^\dagger(k) = \frac{v_g k - \Delta_{\text{C}} - g^2/(v_g k) - i\kappa/2}{v_g k - \Delta_{\text{C}} - g^2/(v_g k) + i\kappa/2}\hat{a}^\dagger(k), \tag{13}$$

where $\hat{a}(k)$ is the Fourier Transform of $\hat{a}(x)$. We note that because there is no loss in the system $|t_k| = 1$.

The two-photon scattering matrix is given by [11, 12] and maps

$$\int dp_1 dp_2 \hat{a}^\dagger(p_1)\hat{a}^\dagger(p_2)|0\rangle f(p_1) f(p_2) \to \int dk_1 dk_2 \hat{a}^\dagger(k_1)\hat{a}^\dagger(k_2)|0\rangle t_{k_1} t_{k_2} f(k_1) f(k_2) +$$
$$\frac{i\sqrt{\kappa}g}{\pi}\int dk_1 dk_2 \hat{a}^\dagger(k_1)\hat{a}^\dagger(k_2)|0\rangle s^a_{k_1} s^a_{k_2} \int dp_1 dp_2 f(p_1) f(p_2)\delta(E_p - E_k)\frac{2g(s^c_{p_1} + s^c_{p_2}) + (E_p - 2\omega_c + i\kappa)(s^a_{p_1} + s^a_{p_2})}{(E_p - \lambda_+)(E_p - \lambda_-)}, \tag{14}$$

where $E_p = p_1 + p_2$ and $E_k = k_1 + k_2$ denote the total input and output wavenumbers which must be equal due to

conservation of energy, and

$$
\begin{aligned}
s_k^a &= \frac{\sqrt{\kappa}\, g}{(v_g k - \Delta_{\mathrm{C}} + i\kappa/2) v_g k - g^2}, \\
s_k^c &= \frac{\sqrt{\kappa}\, v_g k}{(v_g k - \Delta_{\mathrm{C}} + i\kappa/2) v_g k - g^2}.
\end{aligned} \tag{15}
$$

In addition, we have

$$
\lambda_\pm = \frac{2(\omega_0 + \omega_c) + \Delta_{\mathrm{C}} - 3i\kappa/2}{2} \pm \sqrt{\left(\frac{\Delta_{\mathrm{C}} - i\kappa/2}{2}\right)^2 + 2g^2}. \tag{16}
$$

We use (14) to compute the full two-photon output wavepacket in the main text. The unnormalized second-order correlation function is, in the low-input power limit, proportional to the two-photon wavefunction

$$
G^{(2)}(x_1, x_2) = \bar{n}^2 |\psi_2(x_1, x_2)|^2 + O(\bar{n}^3). \tag{17}
$$

Here, $\psi_2(x_1, x_2)$ is the two-photon wavefunction expressed in real space and $O(\bar{n}^3)$ indicates terms of order $\bar{n}^3$ and higher.

1. *Single-photon scattering, delay, and dispersive effects*

With the single-photon transmission coefficient one can calculate the delay and dispersion the cavity QED system imparts on an incoming Gaussian single-photon pulse. For an input state

$$
|\mathrm{in}\rangle = \frac{1}{\sqrt{\sigma}\pi^{1/4}} \int dx \hat{a}^\dagger(x)|0\rangle e^{ik_0 x} e^{-\frac{x^2}{2\sigma^2}}, \tag{18}
$$

we calculate the output state by taking its Fourier Transform, computing the single-photon scattering using (13), and then inverse Fourier Transforming back to real-space. From this we get the output state

$$
|\mathrm{out}\rangle = \sqrt{\frac{\sigma}{2\pi}} \frac{1}{\pi^{1/4}} \int dx \hat{a}^\dagger(x)|0\rangle \int dk\, t_k e^{-(k-k_0)^2\sigma^2/2} e^{ikx}. \tag{19}
$$

The full output state is computed by calculating the Fourier integral over k numerically.

We get analytic insight into the delay and dispersive properties of pulse propagation by Taylor expanding the transmission coefficient about the detuning $\Delta_{\mathrm{L}} = v_g k_0$ at which the Gaussian pulse is centered. Noting that $|t_k| = 1$, we write $t_k = e^{i\phi(\Delta_{\mathrm{L}}))}$, which implies

$$
\phi(\Delta_{\mathrm{L}}) = -i \log(t_k). \tag{20}
$$

We then consider a Taylor expansion of $\phi(\Delta_{\mathrm{L}})$. Keeping terms up to third order in the Taylor expansion we obtain for the output state

$$
|\mathrm{out}\rangle \sim \sqrt{\frac{\sigma}{2\pi}} \frac{1}{\pi^{1/4}} \int dx \hat{a}^\dagger(x)|0,0,g\rangle \int dk\, e^{i\left[\phi(\Delta_{\mathrm{L}}) + (k-k_0)\phi'(\Delta_{\mathrm{L}}) + \frac{(k-k_0)^2}{2}\phi''(\Delta_{\mathrm{L}}) + \frac{(k-k_0)^3}{3!}\phi'''(\Delta_{\mathrm{L}})\right]} e^{-(k-k_0)^2\sigma^2/2} e^{ikx} \tag{21}
$$

$$
= \sqrt{\frac{\sigma}{2\pi}} \frac{1}{\pi^{1/4}} \int dx \hat{a}^\dagger(x)|0,0,g\rangle e^{i(\phi(\Delta_{\mathrm{L}}) - k_0\phi'(\Delta_{\mathrm{L}}))} \int dk e^{ik[x + \phi'(\Delta_{\mathrm{L}})]} e^{-\frac{1}{2}(k-k_0)^2\left(\sigma^2 + i\phi''(x)\right)} e^{i\frac{(k-k_0)^3}{3!}\phi'''(\Delta_{\mathrm{L}})}. \tag{22}
$$

The different derivatives therefore affect the Gaussian pulse in different ways. The first derivative $\phi'(\Delta_{\mathrm{L}})$ describes the delay the pulse undergoes, while the second derivative $\phi''(\Delta_{\mathrm{L}})$ describes the broadening and chirp of the Gaussian pulse. These two terms preserve the Gaussian shape of the pulse. On the other hand the third derivative $\phi'''(x)$ describes pulse distortion, whereby the pulse becomes non-Gaussian in shape. These derivatives can be computed

analytically using the definition of t_k in (13). The first and second derivatives as a function of detuning $\Delta_{\rm L}$ are,

$$\phi'(\Delta_{\rm L}) = \frac{4\kappa(4\Delta_{\rm L}^2 + \Gamma\kappa)}{16\Delta_{\rm L}^4 + \Gamma^2\kappa^2 + 4\Delta_{\rm L}^2\kappa(\kappa - 2\Gamma)}, \tag{23}$$

$$\phi''(\Delta_{\rm L}) = -\frac{32\Delta_{\rm L}\kappa[16\Delta_{\rm L}^4 + 8\Gamma\Delta_{\rm L}^2\kappa + \Gamma\kappa^2(\kappa - 3\Gamma)]}{(16\Delta_{\rm L}^4 + \Gamma^2\kappa^2 + 4\Delta_{\rm L}^2\kappa(\kappa - 2\Gamma))^2)}. \tag{24}$$

Here we have defined $\Gamma = 4g^2/\kappa$. On resonance, when $\Delta_{\rm L} = 0$, the derivatives simplify to

$$\phi'(0) = \frac{4}{\Gamma}, \tag{25}$$

$$\phi''(0) = 0, \tag{26}$$

$$\phi'''(0) = -\frac{32}{\Gamma^3} + \frac{96\Gamma}{\Gamma^3\kappa}, \tag{27}$$

where even-order derivatives are zero due to symmetry. These give significant insight into the role of the cavity. They show that on resonance, given that Γ is fixed, the decay rate of the cavity does not influence the delay imparted on the pulse because $\phi'(0)$ is independent of κ. The delay here is the same as scattering off a bare two-level system [13]. On the other hand, the cavity can significantly reduce the pulse distortion term on resonance $\phi'''(0)$. In particular when $\kappa = 3\Gamma$ the pulse distortion term vanishes. This occurs near the onset of strong coupling where the fluorescence spectrum develops a flat top.

In this section we have taken advantage of the fact that, due to conservation of energy, the plane-wave states created by $\hat{a}^\dagger(k)$ are scattering eigenstates with eigenvalue t_k. We can thus use this to project an input state on a complete basis of these scattering eigenstates and compute the delay and dispersion imparted by the scattering process. Unfortunately, with the form of the two-photon scattering matrix in (14) we do not have the eigenstates and we therefore cannot do this for the two-excitation states. In order to perform an equivalent calculation for the two-photon input states we find the two-photon scattering eigenstate.

C. Two-photon Scattering Eigenstates

We now consider the two-excitation eigenstates. These are significantly more complicated than the plane-wave single-excitation eigenstates. This is because the energy of each individual photon is not necessarily conserved. We follow a process similar to that presented in [14, 15]. The technique is reminiscent of computing two-excitation Bethe states in spin chains [16].

We begin by writing an Ansatz that spans the two-excitation sector,

$$|\psi_2\rangle = \left\{ \frac{1}{\sqrt{2}} \int dx_1 dx_2 \alpha(x_1, x_2) \hat{a}^\dagger(x_1)\hat{a}^\dagger(x_x) + \int dx_1 \hat{a}^\dagger(x_1)\hat{\sigma}_+ b(x_1) + e\hat{\sigma}_+\hat{c}^\dagger + \int dx_1 \hat{a}^\dagger(x_1)\hat{c}^\dagger c(x_1) + c_0 \frac{c^\dagger c^\dagger}{\sqrt{2}} \right\} |0, 0, g\rangle \tag{28}$$

where $|0, 0, g\rangle$ is the state corresponding to no photons in the waveguide or cavity and the atom in the ground state. Using the Hamiltonian in (9), Schrödinger's equation produces five coupled equations

$$-iv_g \left(\frac{\partial}{\partial x_1} + \frac{\partial}{\partial x_1} \right) \alpha(x_1, x_2) + \frac{\eta}{\sqrt{2}} [c(x_1)\delta(x_2) + c(x_2)\delta(x_1)] = E\, \alpha(x_1, x_2), \tag{29}$$

$$2\Delta_{\rm C} c_0 + \sqrt{2}\eta c(0) + \sqrt{2} g e = E c_0, \tag{30}$$

$$-iv_g \frac{d}{dx} b(x) + \eta e \delta(x) + g c(x) = E b(x), \tag{31}$$

$$\Delta_{\rm C} e + \eta b(0) + \sqrt{2} g c_0 = E e, \tag{32}$$

$$-iv_g \frac{d}{dx} c(x) + \Delta_{\rm C} c(x) + \frac{\eta}{\sqrt{2}} [\alpha(0, x) + \alpha(x, 0)] + \sqrt{2}\eta\delta(x) c_0 + g b(x) = E c(x). \tag{33}$$

Our aim is to solve for the scattering eigenstates, which means we want $\alpha(x_1, x_2)$ in the limits $x_1, x_2 \to -\infty$ and $x_1, x_2 \to \infty$, giving the in and out states respectively. We note that due to bosonic symmetry $\alpha(x_1, x_2) = \alpha(x_2, x_1)$.

We follow the standard approach for a two-body problem where we use center-of-mass $x_c = (x_1 + x_2)/2$ and

difference $x = x_1 - x_2$ coordinates. Using these coordinates, when x_1 and x_2 are away from the origin, Eq. 29 simply becomes

$$-iv_g \frac{\partial}{\partial x_c}\alpha(x_c, x) = E\alpha(x_c, x). \tag{34}$$

This has the solution

$$\alpha(x_c, x) = e^{i\frac{E}{v_g}x_c} f(x). \tag{35}$$

The center-of-mass evolution follows the form of a plane wave which results from the two-photon energy being a conserved quantity. The physics of the two-photon scattering process therefore lies in the function $f(x)$.

The function $f(x)$ is piecewise continuous, but has discontinuities in its derivative at $x_1 = 0$ and at $x_2 = 0$. Due to bosonic symmetry $f(-x) = f(x)$, therefore, without loss of generality, we restrict ourselves to $x_1 > x_2$, which implies $x > 0$. Values for $x < 0$ can be obtained via symmetry. Using this information we write an Ansatz

$$f(x) = \begin{cases} f_{\text{in}}(x) & 0 > x_1 > x_2 \\ f_0(x) & x_1 > 0 > x_2 \\ f_{\text{out}}(x) & x_1 > x_2 > 0. \end{cases} \tag{36}$$

With this Ansatz we aim to obtain an equation for $f_0(x)$ in terms of $f_{\text{in}}(x)$ and then an equation for $f_{\text{out}}(x)$ in terms of $f_0(x)$. To get the former we first integrate Eq. 29 from $x_1 = -\epsilon$ to $x_1 = \epsilon$ giving,

$$-iv_g\left(\alpha(0^+, x_2) - \alpha(0^-, x_2)\right) + \frac{\eta}{\sqrt{2}}c(x_2) = 0 \tag{37}$$

$$c(x) = \frac{iv_g\sqrt{2}}{\eta}\left[f_0(-x) - f_{\text{in}}(-x)\right] e^{iEx/(2v_g)}. \tag{38}$$

We then substitute Eq. 33 in Eq. 31 to eliminate $b(x)$, while noting that in these equations we are in the domain $x < 0$. We also make use of the fact that $\alpha(x_1, x_2)$ has bosonic symmetry and is piecewise continuous, i.e.,

$$\alpha(x, 0) = \alpha(0, x) = \left[\alpha(0^-, x) + \alpha(0^+, x)\right]/2 = \left[f_{\text{in}}(-x) + f_0(-x)\right] e^{iEx/(2v_g)}, \tag{39}$$

allowing us to eliminate $c(x)$. After some manipulation, we obtain the differential equation

$$f_0''(x) - af_0'(x) + bf_0(x) = f_{\text{in}}''(x) - \tilde{a}f_{\text{in}}'(x) + \tilde{b}f_{\text{in}}(x), \tag{40}$$

where,

$$a = -\frac{i}{v_g}\left[E - \Delta_{\text{C}} + \frac{i\kappa}{2}\right], \tag{41}$$

$$b = \frac{E}{2v_g^2}\left[\Delta_{\text{C}} - \frac{E}{2} + \frac{2g^2}{E} - i\frac{\kappa}{2}\right], \tag{42}$$

$$\tilde{a} = -\frac{i}{v_g}\left[E - \Delta_{\text{C}} - \frac{i\kappa}{2}\right], \tag{43}$$

$$\tilde{b} = \frac{E}{2v_g^2}\left[\Delta_{\text{C}} - \frac{E}{2} + \frac{2g^2}{E} + i\frac{\kappa}{2}\right]. \tag{44}$$

We have again defined the cavity relaxation rate as $\kappa = \eta^2/v_g$.

We now follow the same procedure to obtain an equation for $f_{\text{out}}(x)$ in terms of $f_0(x)$. We first integrate Eq. 29 from $x_1 = -\epsilon$ to $x_1 = \epsilon$. This gives

$$c(x) = \frac{i\sqrt{2}v_g}{\eta}\left[f_{\text{out}}(x) - f_0(x)\right] e^{iEx/(2v_g)} \tag{45}$$

We then substitute Eq. 33 in Eq. 31 to eliminate $b(x)$, but now we work in the domain $x > 0$. This time we make use

of

$$\alpha(x,0)=\alpha(0,x)=\left[\alpha(x,0^-)+\alpha(x,0^+)\right]/2=\left[f_0(x)+f_{\rm out}(x)\right]e^{iEx/(2v_g)}, \tag{46}$$

allowing us to eliminate $c(x)$. After some manipulation, we obtain the differential equation

$$f''_{\rm out}(x)+af'_{\rm out}(x)+bf_{\rm out}(x)=f''_0(x)+\tilde{a}f'_0(x)+\tilde{b}f_0(x). \tag{47}$$

We now have two coupled second-order differential equations. By operating on Eq. 47 with $\left[d^2/dx^2-ad/dx+b\right]$ we obtain a single differential equation

$$\left[\frac{d^4}{dx^4}+(2b-a^2)\frac{d^2}{dx^2}+b^2\right]f_{\rm out}(x)=\left[\frac{d^4}{dx^4}+(2b-a^2)\frac{d^2}{dx^2}+b^2\right]f_{\rm in}(x)+c\frac{d^2f_{\rm in}}{dx^2}+d\,f_{\rm in}(x), \tag{48}$$

where,

$$c=\frac{2i\kappa}{v_g}\left[\Delta_{\rm C}-\frac{E}{2}\right] \tag{49}$$

$$d=i\frac{E^2\kappa}{2v_g^4}\left[\Delta_{\rm C}-\frac{E}{2}+\frac{2g^2}{E}\right]. \tag{50}$$

To find the eigenstates we then require $f_{\rm out}(x)=\lambda f_{\rm in}(x)$, where λ is a scattering eigenvalue giving the transmission coefficient of the eigenstate. We then have the homogeneous differential equation

$$\left[\frac{d^4}{dx^4}+X\frac{d^2}{dx^2}+Y\right]f_{\rm in}(x)=0, \tag{51}$$

where,

$$X=2b-a^2-\frac{c}{\lambda-1}, \tag{52}$$

$$Y=b^2-\frac{d}{\lambda-1}. \tag{53}$$

Equation 51 has the general solution

$$f_{\rm in}(x)=Ae^{i\Delta_1x}+Be^{-i\Delta_1x}+Ce^{i\Delta_2x}+De^{-i\Delta_2x}, \tag{54}$$

where,

$$\Delta_1=\frac{\sqrt{X+\sqrt{X^2-4Y}}}{2}, \tag{55}$$

$$\Delta_2=\frac{\sqrt{X-\sqrt{X^2-4Y}}}{2}. \tag{56}$$

We previously interpreted the factor in the exponent of the center-of-mass motion as being proportional to the total two-photon energy. Here the factors Δ_1 and Δ_2 in the exponents are proportional to the difference between the energy of the two photons. Rearranging (55) we can get the scattering eigenvalue λ in terms of Δ_1 and Δ_2. Rearranging both equations in (55) leads to the same functional form for the scattering eigenvalue,

$$\lambda(E,\Delta_1)=1+\frac{d-c\Delta_1^2}{a^2\Delta_1^2+(b-\Delta_1)^2}=\lambda(E,\Delta_2)=1+\frac{d-c\Delta_2^2}{a^2\Delta_2^2+(b-\Delta_2)^2}. \tag{57}$$

This indicates that Δ_1 and Δ_2 are not both independent variables, but that one follows from the other. We will choose to use Δ_1 as the independent variable and define Δ_2 in terms of Δ_1. Solving (57) for Δ_2 gives

$$\Delta_2=\pm\frac{\sqrt{b^2c+a^2d-2bd+d\Delta_1^2}}{\sqrt{c\Delta_1^2-d}}=\pm\frac{\sqrt{4Eg^2\kappa^2+\left[E(E-2\Delta_{\rm C})-4g^2\right]\left[E^3+4g^2(3E-2\Delta_{\rm C})-4\Delta_1^2Ev_g^2\right]}}{2v_g\sqrt{-4Eg^2-(E-2\Delta_{\rm C})(2\Delta_1v_g-E)(E+2\Delta_1v_g)}}. \tag{58}$$

We note that the scattering eigenvalue in Eq. 57 can be rearranged to a more intuitive form,

$$\lambda(E,\Delta_1)=\frac{\frac{E}{2}+v_g\Delta_1-\Delta_{\rm C}-\frac{g^2}{E/2+v_g\Delta_1}-i\frac{\kappa}{2}}{\frac{E}{2}+v_g\Delta_1-\Delta_{\rm C}-\frac{g^2}{E/2+v_g\Delta_1}+i\frac{\kappa}{2}}\frac{\frac{E}{2}-v_g\Delta_1-\Delta_{\rm C}-\frac{g^2}{E/2-v_g\Delta_1}-i\frac{\kappa}{2}}{\frac{E}{2}-v_g\Delta_1-\Delta_{\rm C}-\frac{g^2}{E/2-v_g\Delta_1}+i\frac{\kappa}{2}}=t_{E/(2v_g)+\Delta_1}t_{E/(2v_g)-\Delta_1}, \tag{59}$$

where t_k is the single-photon transmission coefficient from (13) and $E/2\pm v_g\Delta_1$ gives the energy of each of the two photons. The two-photon transmission coefficient is therefore a product of two single-photon transmission coefficients for the energy of the two photons.

In order to proceed we need to use the boundary conditions of the problem to derive the values B/A, C/A, and D/A. To do this we first need to compute $f_0(x)$, which we obtain by solving (40) directly,

$$\begin{aligned}f_0(x)=&\frac{\left[-\Delta_1^2-ia\Delta_1+b+i\kappa\Delta_1/v_g+iE\kappa/(2v_g^2)\right]Ae^{i\Delta_1x}}{b-ia\Delta_1-\Delta_1^2}+\frac{\left[-\Delta_1^2+ia\Delta_1+b-i\kappa\Delta_1/v_g+iE\kappa/(2v_g^2)\right]Be^{-i\Delta_1x}}{b+ia\Delta_1-\Delta_1^2}\\&+\frac{\left[-\Delta_2^2-ia\Delta_2+b+i\kappa\Delta_2/v_g+iE\kappa/(2v_g^2)\right]Ce^{i\Delta_2x}}{b-ia\Delta_2-\Delta_2^2}+\frac{\left[-\Delta_2^2+ia\Delta_2+b-i\kappa\Delta_2/v_g+iE\kappa/(2v_g^2)\right]De^{-i\Delta_2x}}{b+ia\Delta_2-\Delta_2^2}\\=&t_{E/(2v_g)+\Delta_1}Ae^{i\Delta_1x}+t_{E/(2v_g)-\Delta_1}Be^{-i\Delta_1x}+t_{E/(2v_g)+\Delta_2}Ce^{i\Delta_2x}+t_{E/(2v_g)-\Delta_2}De^{-i\Delta_2x}.\end{aligned} \tag{60}$$

In the second line the form of $f_0(x)$ is intuitive: one of the two photons has scattered off the cavity and therefore each term acquires a single transmission coefficient.

1. *Boundary conditions*

We now use Eqs. 29-33 to derive boundary conditions for $f_{\rm in}(x)$. Firstly, Eqs. 30 and 32 are boundary conditions that relate c_0 and $c(0)$ as well as b_0 and $b(0)$. Again, we use $c(0)=\left[c(0^+)+c(0^-)\right]/2$ and $b(0)=\left[b(0^+)+b(0^-)\right]/2$. We obtain two more equations by integrating (31) and (33) over the origin,

$$e=\frac{iv_g}{\eta}\left[b(0^+)-b(0^-)\right], \tag{61}$$

$$c_0=\frac{iv_g}{\sqrt{2}\eta}\left[c(0^+)-c(0^-)\right]. \tag{62}$$

Eliminating c_0 and e then gives two equations

$$(2\Delta_{\rm C}-E)\frac{iv_g}{\sqrt{2}\eta}\left[c(0^+)-c(0^-)\right]+\frac{\eta}{\sqrt{2}}\left[c(0^+)+c(0^-)\right]+\frac{i\sqrt{2}v_gg}{\eta}\left[b(0^+)-b(0^-)\right]=0, \tag{63}$$

$$(\Delta_{\rm C}-E)\frac{iv_g}{\eta}\left[b(0^+)-b(0^-)\right]+\frac{\eta}{2}\left[b(0^+)+b(0^-)\right]+\frac{iv_gg}{\eta}\left[c(0^+)-c(0^-)\right]=0. \tag{64}$$

We now relate these boundary conditions to $f_{\rm in}(x)$ and $f_0(x)$. To do this we use (38) and (45) to obtain $c(x)$ in terms of $f_{\rm in}(x)$ and $f_0(x)$ when $x<0$ and $x>0$ respectively. We then use (31) to obtain $b(x)$ in terms of $c(x)$ and thus in terms of $f_{\rm in}(x)$ and $f_0(x)$ when $x<0$ and $x>0$. Using these we obtain two boundary conditions,

$$\left.\frac{df_{\rm in}}{dx}\right|_{x=0}=0, \tag{65}$$

$$2iv_g\left.\frac{df_0}{dx}\right|_{x=0}=\left[(\lambda+1)iK_1+(\lambda-1)K_3\right]f_{\rm in}(0)+2iK_2f_0(0), \tag{66}$$

where $K_1 = -2(E-\Delta_C)(E/2-\Delta_C)/\kappa + \kappa/2 + 2g^2/\kappa$, $K_2 = 2(E-\Delta_C)(E/2-\Delta_C)/\kappa + \kappa/2 - 2g^2/\kappa$, and $K_3 = 3E/2 - 2\Delta_C$. In terms of the constants A, B, C, and D, these conditions are,

$$\begin{aligned} &i\Delta_1(A-B) + i\Delta_2(C-D) = 0, \\ (A+B+C+D)\left[(\lambda+1)iK_1 + (\lambda-1)K_3\right] &= -2\left[(iK_2 + v_g\Delta_1)t_{E/(2v_g)+\Delta_1}A + (iK_2 - v_g\Delta_1)t_{E/(2v_g)-\Delta_1}B\right. \\ &\quad \left.+(iK_2 + v_g\Delta_2)t_{E/(2v_g)+\Delta_2}C + (iK_2 - v_g\Delta_2)t_{E/(2v_g)-\Delta_2}D\right]. \end{aligned}$$

Here we have used a concise notation with $\lambda = \lambda(E,\Delta)$. These two boundary conditions, along with orthonormality, can be used find a class of extended states. We do not use the extended states in the main text for any of the computations.

2. *Bound States*

The general form of the two-photon eigenstates solution (54) is composed of a linear combination of four exponential functions. When Δ_1 and Δ_2 are real, these lead to plane-wave or spatially extended solutions. We are interested in bound-state solutions where the photons are exponentially localised about the difference coordinate, i.e., solutions where Δ_1 and Δ_2 are imaginary or complex with a positive-values imaginary part for $x > 0$. Here Δ_1 is no longer a quantum number labelling the state meaning the state is only labelled by its total two-photon energy E. For these solutions to remain bounded (not go to infinity), we then require $B = 0$ and $D = 0$. The two boundary conditions (65) and (66) give two coupled equations which can be written in matrix form as $\mathbf{M}\mathbf{v} = \mathbf{0}$. Here, $\mathbf{v} = \begin{pmatrix} A & C \end{pmatrix}^T$. We obtain a condition to find Δ_1 by requiring the determinant of the matrix vanish $\det(\mathbf{M}) = 0$,

$$\frac{2K_2(t_{\Delta_1}\Delta_2 - t_{\Delta_2}\Delta_1) + 2iv_g\Delta_1\Delta_2(t_{\Delta_2} - t_{\Delta_1}) + iK_3(\Delta_1-\Delta_2)(\lambda-1) - K_1(\Delta_1-\Delta_2)(1+\lambda)}{2K_2(t_{-\Delta_2}\Delta_1 - t_{-\Delta_1}\Delta_2) + 2iv_g\Delta_1\Delta_2(t_{-\Delta_2} - t_{-\Delta_1}) - iK_3(\Delta_1-\Delta_2)(\lambda-1) + K_1(\Delta_1-\Delta_2)(1+\lambda)} = 0 \tag{67}$$

Here, we have used the concise notation $t_{\pm\Delta} \equiv t_{E/(2v_g)\pm\Delta}$. The eigenvector with coefficients A and C then corresponds to the eigenvector with zero eigenvalue. In general, the value of Δ_1 that satisfies (67) is complex. Furthermore the value of Δ_2 corresponding to this value of Δ_1 is also in general complex. The transmission coefficient for the bound state is then obtained by substituting the complex value of Δ_1 in (57). Similarly Δ_2 is also obtained by substituting the complex value of Δ_1 in (58). We solve these equations numerically and use a two-dimensional minimization algorithm to find the real and imaginary parts of Δ_1.

The general form of the eigenstate is then

$$\begin{aligned} |B_E\rangle &= \frac{1}{\sqrt{2}}\int dx_1 dx_2 \hat{a}^\dagger(x_1)\hat{a}^\dagger(x_2)|0\rangle B_E(x_c,x) \\ B_E(x_c,x) &= e^{iEx_c/v_g}N\left\{Ae^{-\operatorname{Im}(\Delta_1)|x|}\left[\cos(\operatorname{Re}(\Delta_1)x) + i\,\operatorname{sgn}(x)\sin(\operatorname{Re}(\Delta_1)x)\right]\right. \\ &\quad \left.+Ce^{-\operatorname{Im}(\Delta_2)|x|}\left[\cos(\operatorname{Re}(\Delta_2)x) + i\,\operatorname{sgn}(x)\sin(\operatorname{Re}(\Delta_2)x)\right]\right\} \equiv e^{iEx_c/v_g}B_E(x), \end{aligned} \tag{68}$$

where

$$N = \sqrt{\frac{v_g}{2\pi}}\left\{\frac{|A|^2}{\operatorname{Im}(\Delta_1)} + \frac{|C|^2}{\operatorname{Im}(\Delta_2)} + 4\operatorname{Re}\left[\frac{AC^*}{\operatorname{Im}(\Delta_1) + \operatorname{Im}(\Delta_2) - i(\operatorname{Re}(\Delta_1) - \operatorname{Re}(\Delta_2))}\right]\right\}^{-1/2}. \tag{69}$$

Again, we note that the full expression for $B_E(x)$ is obtained by solving for A and C numerically.

3. *Perturbation approximation*

While the full expression for the bound states can only be obtained numerically, we can perform a perturbative expansion to obtain approximate expressions. We begin by expanding (67) to first order in $1/\kappa$ and solving for Δ_1. By $1/\kappa$ we refer to any term where the numerator has units of frequency and the denominator has the value κ. This means we require κ to be larger than all other frequencies in the system. We obtain,

$$\Delta_1 = \frac{i\Gamma}{2v_g} + \frac{i\Gamma^2}{2v_g\kappa} + O\left(\frac{1}{\kappa^2}\right), \tag{70}$$

and

$$C = -A\frac{\Gamma}{\kappa} + O\left(\frac{1}{\kappa^2}\right). \tag{71}$$

Substituting this into the expression for (58) gives,

$$\Delta_2 = \frac{i\kappa}{2v_g} + i\frac{E^2 - 2\Gamma^2}{4v_g\Gamma} + O\left(\frac{1}{\kappa^2}\right). \tag{72}$$

Combining these together and computing the normalization gives,

$$B_E(x_c, x) = \sqrt{\frac{v_g}{4\pi}} \frac{e^{i\frac{E}{v_g}x_c}}{\sqrt{\frac{\kappa}{\Gamma(\Gamma+\kappa)} + \frac{\Gamma^2}{\kappa^3} - \frac{4\Gamma}{\Gamma^2+\Gamma\kappa+\kappa^2}}} \left[e^{-\frac{\Gamma}{2v_g}(1+\frac{\Gamma}{\kappa})|x|} - \frac{\Gamma}{\kappa}e^{-\frac{\kappa}{2v_g}|x|}\right] + O\left(\frac{1}{\kappa^2}\right). \tag{73}$$

In comparison to the two-photon bound state in waveguide QED [13], the addition of a cavity therefore introduces a new term to the bound state. This term, rather than having a decay length inversely proportional to Γ has a decay length inversely proportional to the cavity decay rate κ. It also has the opposite sign to the other term and therefore the two terms interfere destructively. This causes the cusp at $x = 0$ to smooth out.

Using the perturbative solution of Δ_1, we obtain for the transmission coefficient to leading order

$$t_B(E) = \frac{E(\kappa + 2\Gamma) - 2i\Gamma(\kappa - \Gamma - E^2/\Gamma)}{E(\kappa + 2\Gamma) + 2i\Gamma(\kappa - \Gamma - E^2/\Gamma)} + O\left(\frac{1}{\kappa^2}\right). \tag{74}$$

The delay incurred by a resonant pulse of bound states in the center-of-mass coordinate is then

$$\phi_B'(0) = \frac{1}{\Gamma} + \frac{3}{\kappa} + O\left(\frac{1}{\kappa^2}\right), \tag{75}$$

where $\phi_B(E) = -i\log t_B(E)$. The distortion on resonance is then

$$\phi_B'''(0) = -\frac{1}{2\Gamma^3}\left[1 - \frac{3\Gamma}{\kappa}\right] + O\left(\frac{1}{\kappa^2}\right). \tag{76}$$

As discussed in the main text, we highlight that the distortion of the bound state is significantly smaller than the single-photon distortion.

4. *Two-photon bound-state scattering, delay, and dispersive effects*

To compute the scattered two-photon state in the bound subsector we simply compute

$$|\mathrm{out}\rangle_{\mathrm{bound}} = \int dE\, t_B(E)\, |B_E\rangle\langle B_E|\mathrm{in}\rangle, \tag{77}$$

where the bound eigenstates $|B_E\rangle$ are given in (68). Here, the input state is taken to be a separable state,

$$|\mathrm{in}\rangle = \frac{1}{\sqrt{2}}\int dx_1 dx_2 \hat{a}^\dagger(x_1)\hat{a}^\dagger(x_2)|0\rangle f(x_1)f(x_2), \tag{78}$$

where, as for single photon scattering, the functions $f(x)$ can be the pulse shape given by the laser (which are approximately Gaussian) or the exact Gaussian functions in (12). We compute the output state by calculating the integral over E numerically.

We now show that the delay and the dispersion can be computed in the center-of-mass coordinate. We note that the input state can be written in center-of-mass and difference coordinate, which for a Gaussian input becomes

$$|\mathrm{in}\rangle = \frac{1}{\sqrt{2}}\int dx_1 dx_2 \hat{a}^\dagger(x_1)\hat{a}^\dagger(x_2)|0\rangle \frac{1}{\sigma\sqrt{\pi}} e^{2ik_0x_c}e^{-x_c^2/\sigma^2}e^{-x^2/(4\sigma^2)}. \tag{79}$$

The overlap of the input state and the bound state $\langle B_E|\text{in}\rangle = \nu e^{-(E-2k_0)^2\sigma^2/4}$, where the constant ν is the result of the difference-coordinate integral and its form is not relevant here. The output state can thus be written

$$|\text{out}\rangle_{\text{bound}} = \frac{1}{\sqrt{2}}\int dx_1 dx_2 \hat{a}^\dagger(x_1)\hat{a}^\dagger(x_2)|0\rangle\nu\int dE B_E(x)e^{iEx_c}t_B(E)e^{-(E-2k_0)^2\sigma^2/4} \tag{80}$$

$$\sim \frac{1}{\sqrt{2}}\int dx_1 dx_2 \hat{a}^\dagger(x_1)\hat{a}^\dagger(x_2)|0\rangle\nu B_{2k_0}(x)\int dE e^{iEx_c}t_B(E)e^{-(E-2k_0)^2\sigma^2/4}. \tag{81}$$

The first line of this equation is exact, while in the second line we neglect the energy dependence of the bound-state function in the relative coordinate by taking its zeroth-order Taylor term $B_E(x) \to B_{2k_0}(x)$. This is a good approximation when using the perturbative expression for the bound state (73) which is itself, apart from the plane-wave term, independent of E.

Here the bound-state transmission coefficient in the center-of-mass coordinate plays the same role as the single-photon transmission coefficient in (21). We can therefore find the delay and dispersion in the same way as for the single-photon case by defining

$$\phi_B(k_0) = -i\log\left(t_B(E)\right)|_{E=2v_g k_0} \tag{82}$$

The delay and higher-order dispersion for the two-photon bound state are calculated by taking the first, second and third numerical derivatives of (82) in E.

5. *Extended States*

Although we don't use the extended states in the main text, for completeness, we provide a scheme to obtain the coefficients A, B, C, and D. The extended scattering eigenstates are those with the form (54) where the exponents are imaginary and the field is extended in the relative coordinate x. Here there are two sets of exponents, two with $\pm\Delta_1$ and two with $\pm\Delta_2$. As discussed, we take Δ_1 to be the quantum number of the eigenstate and Δ_2 then depends on Δ_1 according to (58). Depending on the values of Δ_1, E, κ, and g, Δ_2 can either be real or imaginary. Noting that in (58) we are free to define the sign of Δ_2, we always choose this such that when it is imaginary it has a positive imaginary part. In this case, for the solution to remain bounded, we require $D = 0$. We then have two boundary conditions and the requirement that the eigenstate be normalized, which gives three conditions in total. Using the boundary conditions to solve for B and C gives

$$\frac{B}{A} = \frac{(\lambda+1)K_1(\Delta_1-\Delta_2) - 2\Delta_2 K_2 t_{\Delta_1} + 2\Delta_1 K_2 t_{\Delta_2} - i(\lambda-1)K_3(\Delta_1-\Delta_2) + 2i\Delta_1\Delta 2t_{\Delta_1}v_g - 2i\Delta_1\Delta_2 t_{\Delta_2}v_g}{(\lambda+1)K_1(\Delta_1+\Delta_2) + 2K_2(\Delta_2 t_{-\Delta_1} + \Delta_1 t_{\Delta_2}) - i(\lambda-1)K_3(\Delta_1+\Delta_2) + 2i\Delta_1\Delta_2 v_g(t_{-\Delta_1} - t_{\Delta_2})}, \tag{83}$$

$$\frac{C}{A} = -\frac{2\Delta_1((\lambda+1)K_1 + K_2(t_{\Delta_1} + t_{-\Delta_1}) - i(\lambda-1)K_3 - i\Delta_1 v_g(t_{\Delta_1} - t_{-\Delta_1}))}{(\lambda+1)K_1(\Delta_1+\Delta_2) + 2K_2(\Delta_2 t_{-\Delta_1} + \Delta_1 t_{\Delta_2}) - i(\lambda-1)K_3(\Delta_1+\Delta_2) + 2i\Delta_1\Delta_2 v_g(t_{-\Delta_1} - t_{\Delta_2})}, \tag{84}$$

where the compressed notation $t_\Delta \equiv t(E/2 + v_g\Delta)$ has been used.

In addition to being imaginary, Δ_2 can also be real. Here, in general, the constant $D \neq 0$. We therefore have four unknown parameters and only two boundary conditions and one normalization condition. The lack of a boundary condition arises because the system has a degeneracy. In (54) one can interchange Δ_1 and Δ_2 and the solution remains valid and has the same eigenvalue (57). In order to obtain a further constraint we can require that the two degenerate solutions,

$$f_{\text{in}}^{\Delta_1}(x) = A_{\Delta_1}e^{i\Delta_1 x} + B_{\Delta_1}e^{-i\Delta_1 x} + C_{\Delta_1}e^{i\Delta_2 x} + D_{\Delta_1}e^{-i\Delta_2 x}, \tag{85}$$

$$f_{\text{in}}^{\Delta_2}(x) = A_{\Delta_2}e^{i\Delta_2 x} + B_{\Delta_2}e^{-i\Delta_2 x} + C_{\Delta_2}e^{i\Delta_1 x} + D_{\Delta_2}e^{-i\Delta_1 x}, \tag{86}$$

are orthogonal. Here we have used the fact that the function $\Delta_2 = h(\Delta_1)$ in (58) is its own inverse, i.e., $\Delta_1 = h(\Delta_2)$. The orthogonality condition can be achieved by requiring $\Delta_1 > 0$, $\Delta_2 > 0$, and

$$\frac{B_{\Delta_1}}{A_{\Delta_1}} = -\frac{C^*_{\Delta_2}}{D^*_{\Delta_2}}, \tag{87}$$

$$\frac{B_{\Delta_2}}{A_{\Delta_2}} = -\frac{C^*_{\Delta_1}}{D^*_{\Delta_1}}. \tag{88}$$

This then gives a sufficient number of constraints to find all unknowns.

VII. Quantifying the influence of pulse distortion using function moments and cumulants

Much like distribution moments $E[x^n]$, cumulants give information on the shape of a distribution. The first three cumulants are

$$\kappa_1 = E[x], \tag{89}$$
$$\kappa_2 = E[(x - E[x])^2] = E[x^2] - E[x]^2, \tag{90}$$
$$\kappa_3 = E[(x - E[x])^3] = E[x^3] - 3E[x^2]E[x] + 2E[x]^3, \tag{91}$$

which are simply the mean and the second and third central moments. The second and third cumulants therefore quantify the width of a function and its asymmetry respectively. Higher order cumulants differ from central moments, but will not be computed here. We define the n-th order moment of an arbitrary normalised function $\psi(x)$, with $\int dx|\psi(x)|^2 = 1$, to be

$$E[x^n] = \int dx\, x^n |\psi(x)|^2 \tag{92}$$

Here we want to quantify the cumulants for the transmitted single-photon wavefunction $\psi_1(x)$ and the transmitted two-photon bound-state wavefunction evaluated along the diagonal $\tilde{\psi}_2(x,x) = \tilde{\psi}_2(x_c, x = 0)$. Here we use the tilde because the wavefunction is normalised along the diagonal rather over the entire two-dimensional space, i.e., $\tilde{\psi}_2(x,x) = \psi_2(x,x)/\sqrt{\int dx|\psi_2(x,x)|^2}$.

Gaussian wavefunctions have the unique property that the first and second cumulants are $\kappa_1^{\rm in} = \mu$ and $\kappa_2^{\rm in} = \sigma^2/2$ and all other cumulants are zero. For Gaussian input pulses one can also obtain approximate analytic expressions for the cumulants of the output pulse. To show this let us consider a Gaussian single-photon input wavefunction and its Fourier Transform

$$\psi_{\rm in}^1(x) = \frac{1}{\sqrt{\sigma}\pi^{1/4}} e^{-(x-\mu)^2/(2\sigma^2)} e^{ik_0 x} \tag{93}$$
$$\psi_{\rm in}^1(k) = \frac{\sqrt{\sigma}}{\pi^{1/4}} e^{-i(k-k_0)\mu} e^{-(k-k_0)^2\sigma^2/2} \tag{94}$$

For the output pulse one can compute the n-th order moment,

$$E[x^n] = \frac{(-i)^n \sigma}{\sqrt{\pi}} \int dk\, t_k e^{-i(k-k_0)\mu} e^{-(k-k_0)^2\sigma^2/2} \frac{\partial^n}{\partial k^n} \left[t_k^* e^{i(k-k_0)\mu} e^{-(k-k_0)^2\sigma^2/2} \right] \tag{95}$$

which can be used to get the cumulants. As in the main text we can Taylor expand the transmission coefficients about the central frequency of the input pulse up to third order. This gives,

$$E[x^n] \sim \frac{(-i)^n \sigma}{\sqrt{\pi}} \int dk\, e^{-ik(\mu+\phi_1'(k_0))} e^{-k^2(\sigma^2/2 - i\phi_1''(k_0))} e^{ik\phi_1'''(k_0)} \tag{96}$$
$$\times \frac{\partial^n}{\partial k^n} \left[e^{ik(\mu+\phi_1'(k_0))} e^{-k^2(\sigma^2/2 + i\phi_1''(k_0))} e^{-ik\phi_1'''(k_0)} \right]. \tag{97}$$

From this we can compute the cumulants for the output state

$$\kappa_1^{\rm out} = \mu - \phi'(k_0) - \frac{\phi'''(k_0)}{2\sigma^2} \tag{98}$$
$$\kappa_2^{\rm out} = \frac{\sigma^6 + \sigma^2\phi''(k_0)^2 + \phi'''(k_0)^2}{2\sigma^4} \tag{99}$$
$$\kappa_3^{\rm out} = \phi'''(k_0) \frac{\sigma^6 - 3\sigma^2\phi''(k_0)^2 - 2\phi'''(k_0)^2}{2\sigma^6} \tag{100}$$

Equivalent expressions can be obtained for the two-photon bound state cumulants where the single-photon transmission coefficient is replaced with the bound-state coefficient, i.e., $\phi_1(k) \to \phi_B(2k)$, and the width $\sigma \to \sigma/\sqrt{2}$.

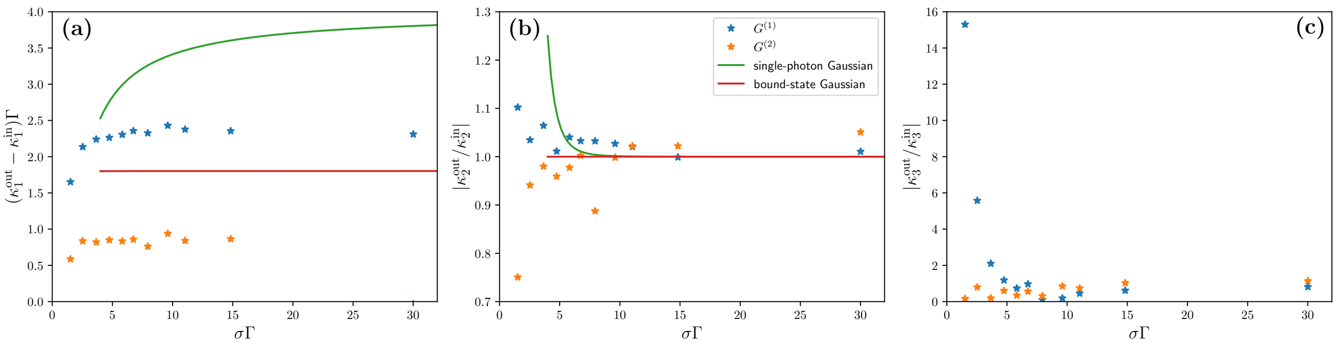

Supplementary Fig. 8. Plot of first to third cumulants versus input pulse width $\sigma\Gamma$. **(a)** Difference between first cumulant of the output and input states quantifies the delay imparted by scattering off the emitter. **(b)** Ratio of second cumulants of the output and input states quantifies the change in width of the pulse. **(c)** Ratio of the third cumulants of the output and input state quantifies the change in asymmetry of the pulse. The lines show the analytic result for Gaussian pulses.

Experimentally, the one- and two-photon bound state wavefunctions are closely related to the transmitted power and equal-time second-order correlation function

$$|\psi_1(x)|^2 \sim \frac{P(x)}{\int dx P(x)} \tag{101}$$

$$|\tilde{\psi}_2(x,x)|^2 \sim \frac{G^{(2)}(x)}{\int dx G^{(2)}(x)} \tag{102}$$

We use the experimental data for the input power and correlation function to compute the input cumulants $\kappa_1^{in}, \kappa_2^{in}, \kappa_3^{in}$ as well as the experimental data for the transmitted field to compute the output cumulants $\kappa_1^{out}, \kappa_2^{out}, \kappa_3^{out}$. This is shown in Fig. 8. Figure 8 shows the difference between the cumulant of the input pulse and the scattered pulse. This quantifies the delay imparted by the emitter on the photons. Clearly the $G^{(2)}$ data records a significantly reduced delay. The variation of the first-order cumulant with pulse width arises due to the influence of distortion. The functional form of this is highlighted from the Gaussian results. The Gaussian results do not agree quantitatively with the experimental data which is expected as the input pulses are not Gaussian. Nevertheless, the analytic results for Gaussian pulses highlight the functional form of the change in delay versus different pulse widths. They also show that the two-photon bound state delay does not vary as strongly with σ. The analytic expressions are for $\sigma\Gamma \gg 1$ and are therefore only plotted for $\sigma\Gamma > 3$.

Figure 8(b) shows the ratio of the second-order cumulants computed for the output and input pulses. This quantifies the ratio of the width of the input and output pulses. For long pulses this approaches unity, but for finite σ the output pulse is broadened. We note that the pulses are resonant so they are ideally not influenced by second-order broadening effects captured by $\phi''(k_0)$. As predicted by the Gaussian analytic results, the single-photon pulses are broadened when the pulse width is reduced. On the other hand the two-photon data tends to show a slight reduction in width.

The ratio of the third-order cumulants is shown in Fig 8(c). This quantifies the degree of asymmetry of the pulse with large values indicating that the output pulse is more asymmetric than the input pulse. The value for Gaussian pulses is not shown as the third order cumulant for an ideal Gaussian input pulse is zero. The plot shows that the $G^{(1)}$ data, which is dominated by the single-photon contribution, becomes significantly more asymmetric after scattering. This does not happen for the $G^{(2)}$ data which either has a similar or reduced asymmetry after scattering.

---

[1] Tomm, N. *et al.* A bright and fast source of coherent single photons. *Nat. Nanotechnol.* **16**, 399–403 (2021).
[2] Tomm, N. *et al.* Tuning the mode splitting of a semiconductor microcavity with uniaxial stress. *Phys. Rev. Applied* **15**, 054061 (2021).
[3] Auffèves-Garnier, A., Simon, C., Gérard, J.-M. & Poizat, J.-P. Giant optical nonlinearity induced by a single two-level system interacting with a cavity in the purcell regime. *Phys. Rev. A* **75**, 053823 (2007).
[4] Antoniadis, N. O. *et al.* A chiral one-dimensional atom using a quantum dot in an open microcavity. *npj Quantum Inf.* **8**, 27 (2022).
[5] Kuhlmann, A. V. *et al.* A dark-field microscope for background-free detection of resonance fluorescence from single

semiconductor quantum dots operating in a set-and-forget mode. *Rev. Sci. Instrum.* **84**, 073905 (2013).
[6] Najer, D. *et al.* A gated quantum dot strongly coupled to an optical microcavity. *Nature* **575**, 622–627 (2019).
[7] Chen, Z., Zhou, Y., Shen, J.-T., Ku, P.-C. & Steel, D. Two-photon controlled-phase gates enabled by photonic dimers. *Phys. Rev. A* **103**, 052610 (2021).
[8] Wigner, E. P. Lower limit for the energy derivative of the scattering phase shift. *Phys. Rev.* **98**, 145–147 (1955).
[9] Bourgain, R., Pellegrino, J., Jennewein, S., Sortais, Y. R. P. & Browaeys, A. Direct measurement of the Wigner time delay for the scattering of light by a single atom. *Opt. Lett.* **38**, 1963–1965 (2013).
[10] Chang, D. E., Sorensen, A. S., Demler, E. A. & Lukin, M. D. A single-photon transistor using nanoscale surface plasmons. *Nat. Phys.* **3**, 807–812 (2007).
[11] Shi, T., Fan, S. & Sun, C. P. Two-photon transport in a waveguide coupled to a cavity in a two-level system. *Phys. Rev. A* **84**, 063803 (2011).
[12] Rephaeli, E. & Fan, S. Few-photon single-atom cavity QED with input-output formalism in fock space. *IEEE Journal of Selected Topics in Quantum Electronics* **18**, 1754–1762 (2012).
[13] Mahmoodian, S., Calajó, G., Chang, D. E., Hammerer, K. & Sørensen, A. S. Dynamics of many-body photon bound states in chiral waveguide QED. *Phys. Rev. X* **10**, 031011 (2020).
[14] Iversen, O. A. & Pohl, T. Strongly correlated states of light and repulsive photons in chiral chains of three-level quantum emitters. *Phys. Rev. Lett.* **126**, 083605 (2021).
[15] Shen, J.-T. & Fan, S. Strongly correlated multiparticle transport in one dimension through a quantum impurity. *Phys. Rev. A* **76**, 062709 (2007).
[16] Giamarchi, T. *Quantum physics in one dimension*, vol. 121 (Clarendon press, 2003).
